# “State of the Mewnion”: Practices of Feral Cat Care and Advocacy Organizations in the United States

**DOI:** 10.3389/fvets.2021.791134

**Published:** 2021-12-14

**Authors:** Sabrina Aeluro, Jennifer M. Buchanan, John D. Boone, Peter M. Rabinowitz

**Affiliations:** ^1^Kitizen Science, Seattle, WA, United States; ^2^Feral Cat Spay/Neuter Project, Lynnwood, WA, United States; ^3^Great Basin Bird Observatory, Reno, NV, United States; ^4^Center for One Health Research, Department of Environmental & Occupational Health Sciences, School of Public Health, University of Washington, Seattle, WA, United States

**Keywords:** free-roaming cat, feral cat, community cat, spay/neuter, trap neuter return, shelter neuter return, return to field, TNR

## Abstract

Over the last several decades, feral cats have moved from the fringes to the mainstream in animal welfare and sheltering. Although many best practice guidelines have been published by national non-profits and veterinary bodies, little is known about how groups “in the trenches” actually operate. Our study sought to address that gap through an online survey of feral cat care and advocacy organizations based in the United States. Advertised as “The State of the Mewnion,” its topics included a range of issues spanning non-profit administration, public health, caretaking and trapping, adoptions of friendly kittens and cats, veterinary medical procedures and policies, data collection and program efficacy metrics, research engagement and interest, and relationships with wildlife advocates and animal control agencies. Respondents from 567 organizations participated, making this the largest and most comprehensive study on this topic to date. Respondents came primarily from grassroots organizations. A majority reported no paid employees (74.6%), served 499 or fewer feral cats per year (75.0%), engaged between 1 and 9 active volunteers (54.9%), and did not operate a brick and mortar facility (63.7%). Some of our findings demonstrate a shared community of practice, including the common use of a minimum weight of 2.0 pounds for spay/neuter eligibility, left side ear tip removals to indicate sterilization, recovery holding times after surgery commonly reported as 1 night for male cats and 1 or 2 nights for females, requiring or recommending to adopters of socialized kittens/cats that they be kept indoor-only, and less than a quarter still engaging in routine testing of cats for FIV and FeLV. Our survey also reveals areas for improvement, such as most organizations lacking a declared goal with a measurable value and a time frame, only sometimes scanning cats for microchips, and about a third not using a standardized injection site for vaccines. This study paints the clearest picture yet available of what constitutes the standard practices of organizations serving feral and community cats in the United States.

## Introduction

Animal shelters across the United States have adopted many intake diversion strategies to decrease the number of relinquished animals, maintain the human-animal bond at all socioeconomic levels, improve life-saving metrics, decrease euthanasia, and to meet community goals. There is a growing body of evidence that feral and community cat advocacy, long term colony management, and trap neuter return (TNR) and shelter-based return-to-field (RTF) programs are effective in keeping cats out of shelter systems, thus reducing feline euthanasia and improving the quality of life for both individual cats and colonies. As an example, a shelter in Albuquerque, New Mexico, started a combined TNR and RTF program that served a total of 11,746 cats over 3 years. During that time, the Albuquerque Animal Welfare Department saw an 84.1% decrease in feline euthanasia and a 37.6% decrease in feline intake (1). Similarly, when a shelter in Jefferson County, Kentucky initiated a RTF program in addition to an existing TNR program, feline euthanasia at the Louisville Metro Animal Services dropped by 94.1% over 8 years (2). This suggests that feral and stray cats may have represented the majority of feline euthanasia performed in these facilities prior to implementing these strategies. A greater understanding of the practices of organizations serving feral cats will help the animal welfare sector standardize and professionalize the care they provide to feral cats. This should lead to further real-world successes and progression of the field.

In addition to concerns around improving animal welfare, there are important One Health aspects of the phenomenon of people interacting with feral cat populations. Cats pose a risk to humans by serving as reservoirs for zoonotic diseases that could be transmitted to individuals who handle or shelter them, as well as act as agents of injury, from minor scratches to bites and more serious infections. Conversely, because of their ongoing exposure to local environments, cats can also serve as valuable sentinels for hazards in the environment that could harm humans. For example, when a large episode of mercury poisoning occurred in communities living near Minamata Bay in Japan in the 1950s, local cats were the first ones to show symptoms because they had greater exposure to contaminated fish and accumulated a toxic level of mercury faster than humans (3). Similarly, cats are studied as sentinels for lead (4), flame retardants (5), chlorinated pollutants (6), and infections such as avian influenza (7). A feral cat presenting as having been poisoned may indicate a risk to people in the area who could also make contact with the substance.

While there has been a growing emphasis in the past decade for animal shelters and rescues to engage in better statistics tracking (8–11), in part at the behest of major funders, these efforts are less well developed among organizations that are not focused on adoptions. Traditionally, groups engaged in TNR programs track the number of surgeries performed, caretaker reports of cats known to frequent certain colonies, and publicly-available data on animal shelter intake and euthanasia. Other types of data and metrics that could be useful for program planning, refinement, and impact assessment, such as the sterilization percentage and density of feral cat populations, tend to be collected and reported much less often.

Quantifying the growth and the impact of the feral cat welfare field without more and standardized data collection practices is a challenge. No previously-published research has sought to study its full extent in the United States, despite some formal documentation of projects dating to the early 1990s (12, 13). In total, 1 metric for charting the popularity of this movement is the financial support it has garnered from the public and grantmaking institutions. This however is complicated by the fact that most organizations that undertake feral cat work also engage in other animal welfare activities. Alley Cat Allies is an exception in that it focuses only on feral cat issues at a national level. In examining their total revenue as reported across the 19 available years of tax returns cataloged online by ProPublica (14), and adjusting these amounts for inflation to August 2021 (15), there has been a clear upward trend. From taking in approximately $3,079,005 USD in fiscal year 2001 (adjusted from $1,988,764 USD) to approximately $11,609,361 USD in fiscal year 2019 (adjusted from $10,905,204 USD), Alley Cat Allies has experienced sizable and steady growth within this century, suggesting that concern and interest in feral cat welfare may be on the rise.

While retrospectively analyzing historical trends isn't always possible, there is always a need for more endeavors aimed at cataloging the field and following its continued refinement moving forward. To address the lack of comprehensive, national scale information about what constitutes a typical community of practice, we conducted an online survey of feral cat care and advocacy organizations based in the United States.

## Materials and Methods

Advertised as “The State of the Mewnion,” our online survey ran from January to March 2018 using the SurveyMonkey.com platform. We cast an inclusive net, asking for participation from organizations of all sizes that self-identified as involved in any aspect of feral cat care and advocacy, without restricting respondents to entities that only worked with such cats. The language we used for participants was “feral and community cats,” but we did not further define those terms. We allowed respondents to use their own judgement of what constitutes a feral or community cat, which may vary slightly among participants. A file containing questions and answer choice options appears in this article's (Supplementary Materials). All questions after the organization demographics section were optional. Most consisted of a set of multiple choice answers, sometimes with the ability to select more than 1 option, and some with the ability to enter a write-in response. Questionnaire topics included non-profit administration and policy, public health, cat caretaking and trapping, adoptions of friendly kittens and cats, veterinary medical procedures and policies, data collection and program efficacy metrics, research engagement and interest, and relationships with wildlife advocates and animal control agencies. Our survey and study protocol were reviewed by the University of Washington's Human Subjects Division as STUDY00004003.

We promoted the survey in Facebook Groups dedicated to feral and community cats, cat rescue, or TNR, invited participation from feline, shelter medicine, and feral cat student clubs at veterinary schools, emailed cat-focused organizations listed on Petfinder's rescue database and the Humane Society of the United States' list of community cat organizations, contacted groups found by searching Google for terms such as “feral cats,” “stray cats,” and “TNR,” and used our personal contact lists. During this process, we also noticed individuals who were not part of the study team sharing the survey on Facebook, increasing our reach organically within these niche communities through social media-based snowball sampling (16). While we attempted to remove duplicate email addresses and avoid reaching out to any group multiple times, it is unknown how many of our contact attempts could have been duplicates or made to an organization which has ceased to operate or did not work with feral cats. Determining a response rate would not be possible.

In addition to gathering descriptive data with the intention of repeating our survey to track sector-wide trends over time, we also sought to explore whether there were organization demographic factors influencing their adherence to popular best practice guidelines produced by major animal welfare and veterinary entities. In the survey, we asked respondents to select which guidelines they used so that we could compare the advice from the 5 most popular answers cited across all organizations. This allowed us to quantitatively define what constitutes popular guidelines rather than using our own judgement, and to test how well respondents are incorporating the advice within. We assumed the recommendations in these guidelines would form a community of practice standard for feral cats, although not necessarily an objectively “correct” standard for all contexts. For example, in private veterinary offices, routine radio-frequency identification microchipping is arguably a best practice. However, in the context of seeking to provide high volume care focused on population reduction and achieving the best outcomes for the greatest number of unowned cats, microchipping is not a priority.

We solicited write-in answers for some items, which we then categorized into groups for reporting results. In total, 1 author tallied responses to the questions asking organizations to list their news/informational resources and TNR/medical best practice resources. For other write-in questions, 2 or 3 authors discussed and reached agreement about how they should be categorized.

To test for associations between best practice adherence and demographics of respondent organizations, we used generalized linear models (GLMs) modeled under a binomial distribution. For our response variables, a respondent's best practice adherence was defined as the number of answers aligned with best practice recommendations (from 0–12) out of a total number completed by the respondent (from 0–12). For our predictor variables, we looked at the following organization-level demographic factors: the Census Region of the United States where an organization is based, the geographic scope covered by the organization, if the organization served urban, suburban, and/or rural areas, the approximate proportion of animals served that were feral cats, whether the organization had its own 501(c)3 United States federal non-profit charity status, whether the organization had a brick-and-mortar facility, the approximate number of feral cats that a respondent served per year, the number of paid employees, and the number of active volunteers. We began with a GLM containing all 11 organization demographic predictors and used the step() function found in R for automated bi-directional model selection based on their Akaike information criterion (AIC). We used the default cut-off criteria for model selection with the step() function, k = 2 or approximately *p* = 0.157.

We performed statistical analysis and created graphics using R (17) with R Studio (18) and the packages plyr (19), plotrix (20), dplyr (21), ggplot2 (22), tidyverse (23), maps (24), alberusa (25), ggthemes (26), and pathwork (27).

## Results

### Demographics and Basics

**Figure 1:**
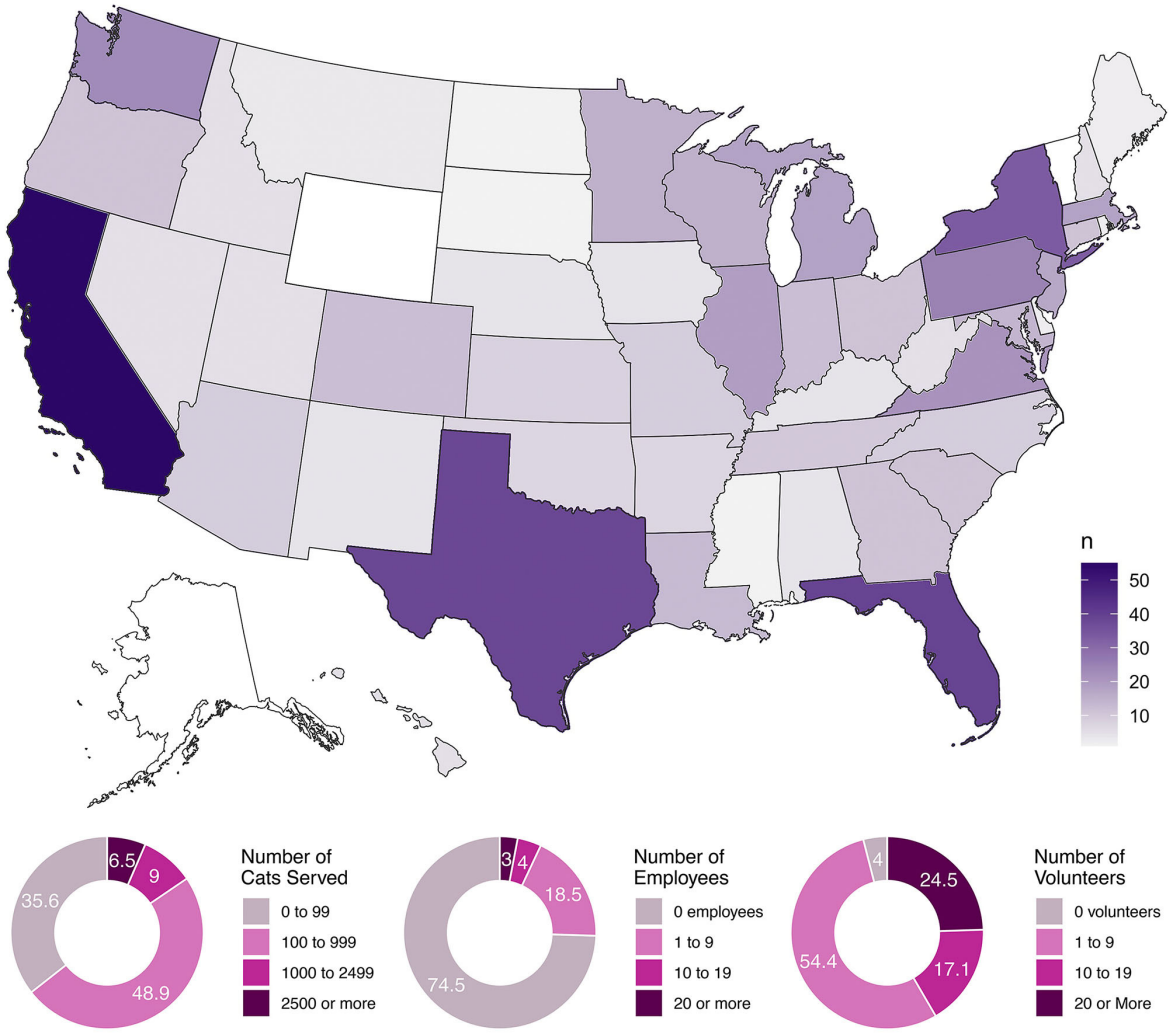
A choropleth map of the United States showing the geographic distribution of our respondent organizations and their basic demographics.

XOur survey received responses from 567 organizations. Our data represented every state except for Alaska, Vermont, and Wyoming, with the populous states of California, Florida, Texas, and New York drawing commensurately high levels of participation (see Figure 1). We used the phrasing “feral/community cats” in our survey questions, but report simply “feral cats” here for brevity.

Respondents came primarily from grassroots organizations, as shown from answers to the required demographic questions (*n* = 567). A majority employed no paid employees (74.6%), reported serving 499 or fewer feral cats per year (75.0%), had between 1 and 9 active volunteers (54.9%), and did not operate a brick and mortar facility (63.7%), although 73.7% did have their own 501(c) federally-recognized non-profit status. In terms of the geographic scope of their activities, 12.0% operated at the level of a neighborhood or development, 72.7% at the level of a city or town, 10.0% statewide, 4.9% multi-state, and 0.4% at a national level. Regarding human density, 68.1% served urban areas, 77.2% served suburban areas, and 70.7% served rural areas (not mutually exclusive). Most of these organizations were not feral cat exclusive, with 44.4% of respondents estimating that three-quarters or more of animals they serve were feral cats, 16.6% estimating between 1 half and three-quarters feral cats, 20.6% reporting one-quarter to 1 half feral cats, and 18.3% reporting 1 quarter or fewer feral cats. In total, 4 respondent groups (0.7%) identified as being projects/clubs operated by veterinary students.

Respondent organizations filled a wide variety of roles across a spectrum from hands-on to policy work (*n* = 567). Among the most popularly-reported primary functions, of which an organization could choose multiple, 53.6% were engaged with the direct feeding and colony care for feral cats, 38.6% socialized/fostered kittens from feral cats for adoption, 31.7% offered low-cost sterilization/vaccination/basic medical care for feral cats, 28.2% offered free sterilization/vaccination/basic medical care for feral cats, and 30.2% coordinated volunteers who are trapping feral cats for TNR. Less common primary functions included 11.6% of organizations reporting that they campaigned for law and policy changes around feral cats, 8.5% operated their own clinic focused on feral cat care, 6.5% engaged in organization-level training and mentorship to other feral cat groups, 4.6% provided disaster relief for feral cats as needed, and 2.6% provided grants and funding organizations doing feral cat work.

When asked if TNR is explicitly allowed or endorsed by local laws and animal control ordinances where they operate, 46.5% of respondents answered yes, 17.8% answered no, 9.9% were unsure, and 25.9% reported that it varies based on the areas in which they work (*n* = 566). For organizations operating where TNR is not explicitly legal, we asked whether there are local laws that could be used, or are actively enforced, to prohibit or limit feral cat care, feeding, or TNR. Of the laws reported to be actively enforced, in descending order or popularity, respondents noted mandatory stray holding periods (171), animal control of nuisance animals (116), pet limits (103), pet licensing laws (73), laws defining outdoor cat feeders as the cat's owner (63), abandonment laws (57), laws against feeding (43), mandatory spay/neuter requirements (33), leash laws which include cats (26), microchipping requirements (20), colony registration requirements (16), and laws restricting veterinarians' abilities to provide free/low-cost services (7). Despite these potential challenges, only a minority of organizations had consulted with an attorney regarding legal problems that could arise from their work. Just 8.2% of respondents were working with an attorney on an ongoing basis, 24.5% having done so in the past, 6.1% were unsure, and 61.1% had not (*n* = 558).

**Figure 2:**
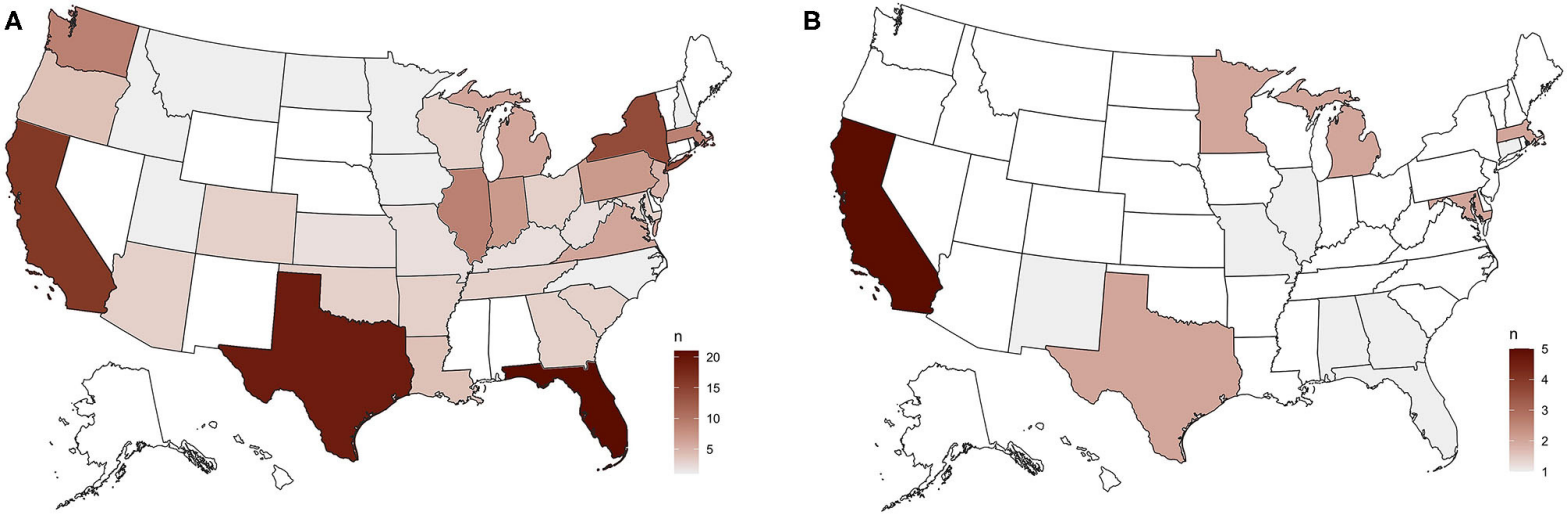
**(A)** Organizations reporting that feral cat advocates and animal control authorities involved in active collaboration were distributed similarly to our overall sample. **(B)** Organizations reporting public/overt conflict between feral cat advocates and animal control authorities, with the greatest number from California.

When describing the relationship between feral cat advocates and animal control authorities in their area, 3.9% felt that public/overt conflict was occurring, 11.0% reported some tension between groups, 16.9% neutral or no interactions, 17.4% some efforts being made toward bridge-building, 32.9% active collaboration and working toward shared goals, 15.6% that they serve many locations and each was different, and 2.3% of respondent groups were themselves the animal control authorities for their area (*n* = 563) (see Figure 2).

In cases where feral cats were only one part of their work, we asked organizations to select reasons they didn't serve more feral cats. The most commonly-selected options were that they would do more with feral cats if they had specific grants/funding (330), they are a comprehensive animal program that fulfilled many roles (197), they didn't have the proper facilities or equipment (182), there was an alternative for feral cat care in their area (53), their staff didn't have the proper training (31), concern about possible injuries to staff and volunteers (15), and that the organization had a policy that prevents (more) care of feral cats (7). Out of the write-in answers to this question, other common themes emerged, with explanations grouped into respondents expressing a need for volunteers (74), a need for personnel/staff (32), a need for spay/neuter services (26), being a small or rural group (24), a need for trappers and places to trap (23), a need for transportation (8), a need for foster homes (8), and limits of partner organizations (8).

In asking respondents a write-in question about which resources (books, websites, blogs, Facebook groups, etc.) they regularly use and trust for updates, information, and news about feral cat issues, the most commonly-cited sources were Alley Cat Allies (223), Best Friends Animal Society (56), Neighborhood Cats (46), the Humane Society of the United States (44), the American Society for the Prevention of Cruelty to Animals (33), Community Cats Podcast (21), Maddies Fund (17), Peter Wolf or his Vox Felina blog (10), and the Million Cat Challenge (9). About a third (37.1%) of organization reported having a locally-focused online discussion group or email list where feral cat advocates can ask questions, share resources, seek assistance, and support 1 another (*n* = 566).

We asked organizations whether they currently had at least 1 declared goal that includes both a measurable value and a timeframe such as “reduce the outdoor cat population of our town 25% by 2025” or “provide 1,000 free spay/neuter surgeries every year.” About 1 third (32.1%) reported that they do, while 67.9% did not (*n* = 563).

### Environmental, Human, and Public Health

When asked if they were seeing health issues in feral cats suspected of being caused by exposures to toxins or environmental contaminants, 13.8% respondents reported yes, 59.0% of reported no, and 27.2% reported that it was unknown (*n* = 544). In categorizing write-in explanations, the common trends for those reporting a concern were chemical or toxic exposures (51), infectious diseases (10), climate and weather related issues (5), reproductive issues and birth defects (4), suspected cancers and carcinogens (4), and firearms (3).

Some respondents suspected observing health issues in feral cats caused by environmental exposures, with the intentional poisoning of cats as the most commonly-mentioned problem. Write-in suspected toxicants and sources of concern included antifreeze, rodenticides, agricultural chemicals, and drinking polluted water. Illnesses mentioned by respondents as presumed to be associated with environmental exposures included infectious disease, cancers, birth defects, eye problems, kidney disease, skin issues, and plasma cell pododermatitis (“pillow pad”), some of which may be linked to environmental factors in cats (28–31). Other responses included cats as victims of hazards in their environment including firearms, flooding, mold, and hurricanes.

The physical and mental health of human participants is another component of feral cat projects. We inquired if organizations maintained insurance for staff and volunteers to cover medical care for injuries sustained during work with feral cats, and only 33.6% responded yes (*n* = 542). When asked if they have a formal process for staff or volunteers who receive bites or other injuries from feral cats, 40.0% groups responded in the affirmative (*n* = 543). We also asked if they provided staff and/or volunteers with mental health care resources, such as information on compassion fatigue, support groups for animal welfare workers, suicide and crisis hotlines, or referrals to mental health providers, and 12.9% reported that they do (*n* = 543).

### Caretaking, Trapping, and Release

Caretaking of feral cats can be done with varying levels of formality and record-keeping. When asked whether colonies or colony caretakers in their service area required by law to be registered in some way, most organizations reported that they are not (76.9%), some were unsure (14.0%), and some reported yes (9.2%) (*n* = 523). Regardless of whether registration is required by law, we asked organizations approximately what proportion of colonies or colony caretakers in their service area did they estimate were actually registered. The vast majority estimated one-quarter or less (86.9%), with 6.7% estimating one-quarter to half, 1.7% estimating half to three-quarters, and 4.8% estimating three-quarters or more (*n* = 480). For those organizations that do register colonies or caretakers, a majority reported that they don't know where that information was stored (57.4%). Of those aware of where the information was stored, 28.8% reported it was with a private non-profit or individual, 6.9% with a government office, and 6.9% stored with both government and private entities (*n* = 378). We asked organizations to select their reasons if they do not always register colonies or caretakers. The most commonly-chosen options, of which they could select multiple, were that they lacked the time or personnel to maintain a registry (157), didn't see a reason to register colonies/caretakers (145), lacked the tools or technical resources to maintain a registry (87), some caretakers had refused (81), they believed caretakers might be resistant (78), feeding/TNR is illegal in their area (52), they were intending on implementing a registry (or better registry) soon (26), and they were advised by an attorney to not document colonies/caretakers (11). Out of write-in answers to this question, we grouped explanations into respondents expressing that they do not register colonies or caretakers because the group has their own records (35), registration is not required (32), fear of how the data could be used (29), noting that there was no way to register (9), and that another entity has a registry (6).

**Figure 3:**
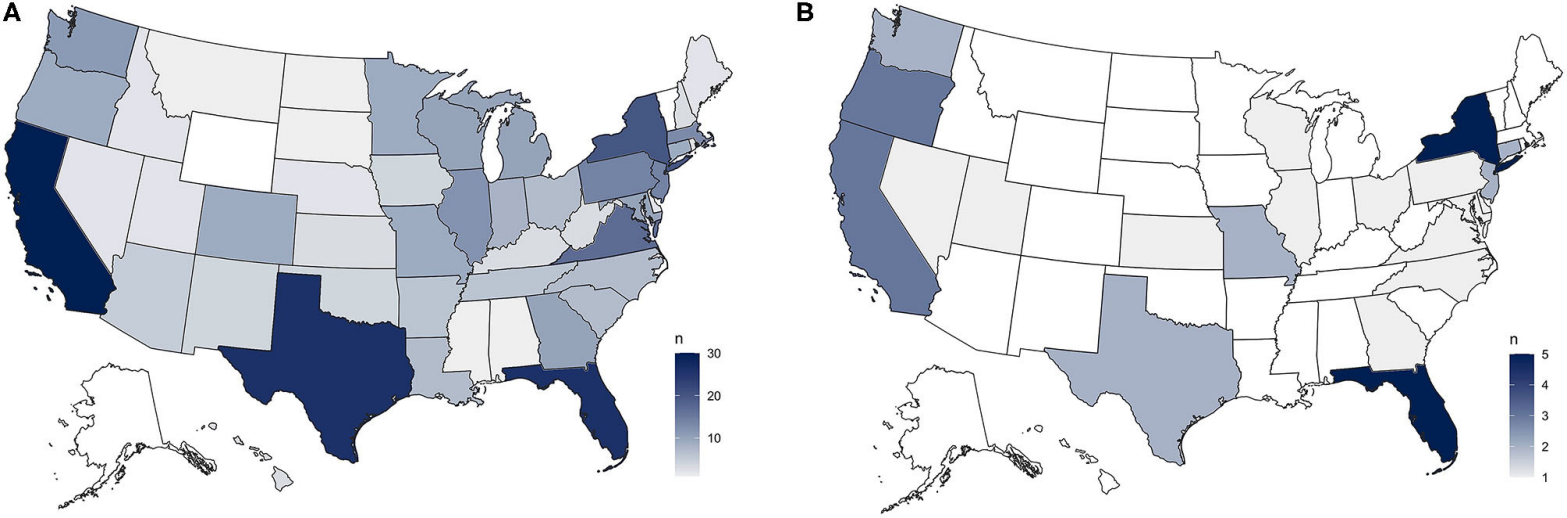
**(A)** In asking organizations how they decided where to trap cats for sterilization, our most common answer was via requests from colony caretakers, which followed a distribution similar to our overall sample. **(B)** Our least common trapping motivation, areas where cats were suspected to pose a risk to birds, was selected the most by organizations from New York and Florida.

For groups trapping feral cats for sterilization, we asked how they decided where to trap, rating a list of options as higher priority, lower priority, or not a factor. The most commonly-selected high priority reasons were requests from colony caretakers (371), trapping in 1 area or colony until all cats were caught and sterilized (364), complaints from the public about the number of cats in a location (335), trapping for TNR and relocation to protect cats at risk of harm (264), providing TNR services to low-income neighborhoods (238), concentrating efforts in smaller areas to get high sterilization coverage of some areas (188), locations from which many cats were entering the shelter/animal control system (188), locations that were safe for trappers to work (174), places located conveniently for trappers (such as near their homes) (151), based on funding/grants that specified where they provide services (136), evenly distributing efforts to provide some sterilizations to as many caretakers as possible (102), and areas where cats were suspected to pose a risk to birds and wildlife (40) (see Figure 3).

Finding homes for kittens was a primary (38.6%) or secondary function of many groups (42.2%) (*n* = 567). For kittens (born to feral cats) under 2 months of age, we asked organizations how commonly they remove them from the outdoors for fostering, socialization, and adoption. Almost half (48.4%) reported that they always do, 28.0% usually, 15.9% sometimes, 5.9% rarely, and 1.8% never (*n* = 510). For kittens (born to feral cats) between 2 and 3 months of age, we see an overall response that shifted away from removal from the outdoors, with 23.5% selecting always, 12.4 % usually, 31.0% sometimes, 29.8% rarely, and 3.3% never (*n* = 510). For organizations that facilitated adoptions of socialized feral cats (as pets, not working/barn cats), we asked if their adoption information had a position on whether these cats should be kept as indoor-only animals. Just over half (52.7%) required that adopted cats/kittens be indoor-only, 28.5% recommended that adopted cats/kittens be indoor-only, 1.2% recommended that adopted cats/kittens be allowed both indoors and outdoors, 9.8% had no position on where adopters keep their cats/kittens, and 7.7% chose “Other” (*n* = 491).

After being trapped and sterilized, organizations tended to hold cats for different lengths of time based on sex. After a routine neuter with no complications, 6.7% released males on the same day as surgery, 73.9% held males overnight before release, 9.2% held males for 2 nights before release, 2.9% held males for 3 nights before release, and 7.3% selected “Other” (*n* = 510). After a routine spay with no complications, 2.7% released females on the same day as surgery, 47.9% held females overnight before release, 24.1% held females for 2 nights before release, 13.0% held females for 3 nights before release, and 12.3% selected “Other” (*n* = 514).

We asked organizations if they routinely recommended or used any supplements or alternative medicine products with feral cats, and if so, to select which type(s). Some organizations answered “no” to this question but selected 1 or more types. By re-coding some “no” responses so that organizations reporting use of specific modalities were tallied as a “yes,” 57.0% of respondents did not routinely recommend or use alternative medicine, whereas 43.0% did (*n* = 567). Commonly-reported were probiotics such as FortiFlora (used by 17.1% of respondents), Feliway pheromone spray (14.5%), Rescue Remedy flower essence (9.2%), homeopathic products (6.7%), herbal products (4.2%), and glucosamine (3.2%).

### Clinical and Medical Issues

Most of our respondent organizations were small projects, and as such, would likely not have a staff veterinarian. For organizations that trapped cats but did not operate a clinic, we asked approximately how far animals must be transported to reach their nearest provider of sterilization services for feral cats. About half (54.5%) were able to reach such a provider in under 30 min by car, 38.9% required 30–60 min, 4.7% required 60–90 min, and 1% apiece required 90–120 min and 2–4 h by car (*n* = 404). Regardless of whether or not they operated their own clinic, we also asked approximately how far away was the next-nearest provider of sterilization services for feral cats. These driving distances did not change greatly, as 46.4% were able to reach a second option in under 30 min, 41.5% required 30–60 min, 7.8% required 60–90 min, 2.8% required 90–120 min, and 1.5% required 2–4 h by car (*n* = 463).

Costs, as well as transportation time, is another issue for accessing veterinary care. For organizations that provided or facilitated sterilization and veterinary services, we asked whether their fees were different for cats reported as owned vs. cats reported as being feral cats, with 56.4% reporting yes and 43.6% reporting no (*n* = 328). For organizations that provided free or discounted services to low-income caretakers and trappers, we asked if they had a stated cut-off for what qualifies as “low-income”. Over half (64.1%) did not, 11.6% did state a cut-off, and 24.3% decided on a case-by-case basis (*n* = 251). For those that did use a cut-off, we asked if they required documentation of low-income status, such as a pay stub, tax return, or qualification for federal assistance programs like Medicare. The vast majority (84.6%) did not ask for such documentation, although 15.4% did (*n* = 311).

Among organizations that had a required fee or suggested donation for feral cats, we asked the amount for 6 common types of basic services. (Some respondents entered $0.00 in response; we dropped zeros from calculations since this question was about fees.) The mean fee or suggested donations for a routine female spay was $44.58 (SD $22.63, range $10.00–120.00), routine male neuter $37.72 (SD $18.89, range $10.00–115.00), routine female spay plus rabies vaccine $48.06 (SD $24.54, range $5.00–130.00), routine male neuter plus rabies vaccine $42.24 (SD $20.60, range $5.00–130.00), routine female spay plus rabies and FVRCP vaccines $53.83 (SD $29.24, range $5.00–195.00), routine male neuter plus rabies and FVRCP vaccines $48.73 (SD $25.38, range $5.00–158.00).

**Figure 4:**
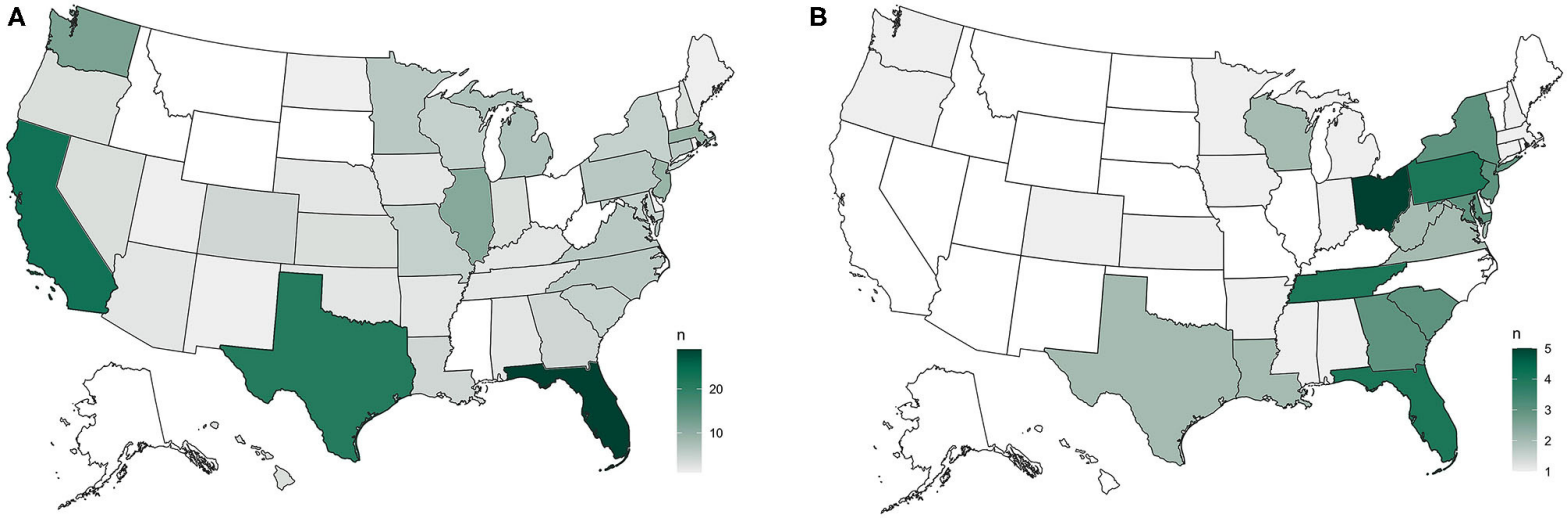
**(A)** Just over half of our respondents reported always scanning cats for identification microchips during their TNR process, with a distribution similar to our sample as a whole. **(B)** There was a regional trend among organizations that reported never scanning cats for microchips, revealing this practice is largely an East Coast phenomenon.

Regarding identification microchips, 52.6% of respondents reported that they always scan feral cats for microchips during their TNR process, with 34.6% reporting that they sometimes do, and 12.8% never scanning for microchips (*n* = 439) (see Figure 4). For organizations that microchip feral cats, the information was registered with different entities. The most commonly-selected answer options indicated that the chips were registered with a standard pet microchip company's database (114), registered with a rescue group (73), registered with local animal control (29), and 28 respondents noted that chip numbers were just for the caretaker's records.

To build a picture of what comprises typical veterinary care offered to feral cats besides just sterilization surgery, we asked organizations which services were part of their process and to categorize them as routine (done to every animal), done at the discretion of a veterinarian or vet tech, provided if requested by a caretaker, or not offered. Described here as a count, the percentage rating that service as routine, and number of respondents answering about that service, services most commonly considered routine included rabies vaccination (383, 89.5%, *n* = 428), FVRCP vaccination (245, 59.0%, *n* = 415), flea/ectoparasite treatment (215, 50.5%, *n* = 426), meloxicam or other injectable pain relief (185, 44.7%, *n* = 414), and de-worming/endoparasite treatment (171, 40.8%, *n* = 419). Services less commonly considered routine included FeLV testing (95, 23.2%, *n* = 412), FIV testing (89, 21.9%, *n* = 407), microchipping (77, 19.1%, *n* = 403), and fluids (46, 11.3%, *n* = 407).

While only a minority of organizations routinely test for feline immunodeficiency virus (FIV) and feline leukemia virus (FeLV), about half of respondents reported offering testing at caretaker request or perform testing at the discretion or a veterinarian or technician. Our survey explored how groups act upon test results. If a feral cat has a positive FeLV test, the most common recommendation options for the cat were retesting at a later date (91), retesting on serum (72), euthanasia if the cat is symptomatic (48), transferred to a rescue/shelter (28), returning to site after sterilization (25), and euthanasia regardless of symptoms (22). Of the write-in answers, we grouped new response types into 4 categories: organizations tried to adopt/foster the cat (16), transfer/relocate the cat (16), isolate the cat (5), or monitor the cat (2). If a feral cat has a positive FIV test, the most common recommendation options for the cat were returning to site after sterilization (95), euthanasia if the cat is symptomatic (68), transferred to a rescue/shelter (41), euthanasia regardless of symptoms (37), retesting at a later date (31), or retesting on serum (7). Of the write-in answers, we grouped new response types into 4 categories: organizations tried to adopt/foster the cat (21), monitor the cat (6), transfer/relocate the cat (5), or isolate the cat (3).

For vaccinations, two-thirds of organizations reported using a standard vaccine injection site. Most commonly, 42.0% gave rabies in the right rear leg and FVRCP in the right front leg, 17.6% gave rabies in the right rear leg, 7.4% used some other standard location, and 33.1% had no standard location for vaccines (*n* = 393). In asking organizations how often they re-trap cats in managed/cared-for colonies for rabies re-vaccination, 1.6% reported doing so always, 4.9% usually, 17.8% sometimes, 25.0% rarely, and 50.7% never (*n* = 428).

Feral cats receive some type of permanent marking to indicate them as sterilized after a spay/neuter surgery. We asked organizations to rate 4 methods as being performed always, on request, or never, and the options aren't mutually exclusive. The most common answers were always using ear tipping (removal of the tip of the ear under anesthesia) (408 respondents), always placing a tattoo in ventral midline abdominal region (152), always using ear notching (removal of a notch from ear) (25), and always placing an ear tattoo (15). There were also write-in answers revealed that 2 organizations reported the use of microchipping and 3 reported tattooing females. Of organizations using ear tipping or notching on of feral cats, 64.8% did so on the left side, 16.4% on either side, 12.7% on the right side, 5.2% did the right side for females and left for males, and 0.9% did the right side for males and left for females (*n* = 440).

For kitten spay/neuter, 58.1% use a minimum weight, 3.5% use a minimum age, and 38.3% require kittens to meet both a set age and weight (*n* = 454). Among organizations that use only a weight, the most common answers were 2.0 pounds (182 respondents), 3.0 pounds (45), 2.5 pounds (15), and 4.0 pounds (10). Only 14 organizations reported a weight less than 2.0 pounds, with 1.6 pounds being the lowest reported minimum weight. Among organizations using only an age, the most common answers were 8 weeks or 2 months (35 respondents), 12 weeks or 3 months (18), 16 weeks or 4 months (12). In total, 3 organizations reported a minimum of 5 weeks as the lowest age limit. Finally, among organizations using both a weight and age, the most common answers were 2 pounds and 2 months (75 respondents), 3 pounds and 3 months (32), and 2 pounds and 3 months (11). The lowest minimum reported for this option was 2 organizations using 2.2 pounds and 2 months.

In describing typical recovery care offered to feral cats after surgery, we found that standard processes after routine surgery often include a small amount of food provided after patient is sternal and alert (209), heat support (147), checking a patient's respiratory rate (130), checking a patient's heart rate (119), checking a patient's mucous membranes/capillary refill (107), checking a patient's temperature (95), corn syrup or dextrose applied along the gumline/mouth (50), administration of subcutaneous fluids (46), and administration of subcutaneous fluids in females only (25). Slightly more organizations reported a single-stage recovery process where a cat is immediately placed in its carrier/trap after surgery (145) than reported a two-stage recovery process where a cat is first attended to outside of a carrier/trap, then placed into a carrier/trap as the cat regains consciousness (131).

When asked whether perioperative antibiotics were part of a routine spay/neuter procedures, a majority of organizations reported that they are not (71.7%), but a sizable minority selected yes (28.3%) (*n* = 381). When using antibiotics for any condition, we asked which types of antibiotics organizations used, with the option to select multiple. Veterinary-formulated/marketed antibiotics were most commonly-reported (379), followed by fish/aquarium-formulated/marketed (51), human-formulated/marketed (46), and antibiotics available in feed stores for farm animals (33).

For organizations that are private non-profits, we asked if they currently received assistance (financial or supplies) from government public health or animal control programs. A large majority reported no (88.0%), with 9.9% reporting yes and 2.1% unsure (*n* = 434). Among organizations answering yes, we inquired about the form of that assistance, allowing for multiple answers. The most common answers included grants and general financial help (27), animal control contracts (9), spay/neuter services (7), rabies vaccines for cats (7), FVRCP vaccines (5), humane traps and animal capture supplies (3), drugs or surgical supplies and equipment (3), and vouchers/reimbursements (3).

Humane live outcomes aren't always possible. We asked organizations to select conditions for which humane euthanasia would be recommended in feral cats. The most common conditions chosen were signs of chronic illness (177), masses suspected of being neoplastic (172), a single FeLV positive test if cat is symptomatic (135), severe respiratory disease (106), a single FeLV and FIV positive test if cat is symptomatic (103), a single FeLV and FIV positive test if cat is symptomatic (103), multiple FeLV positive tests if cat is symptomatic (99), feline stomatitis or severe dental disease (93), a single FIV positive test if cat is symptomatic (85), loss of vision (81), a single FeLV positive test regardless of symptoms (80), multiple FIV positive tests if cat is symptomatic (53), a single FeLV and FIV positive test regardless of symptoms (45), loss of limb (44), multiple FeLV positive tests regardless of symptoms (39), feline plasma cell pododermatitis (37), a single FIV positive test regardless of symptoms (32), cannot return to previous location (26), heart murmur or arrhythmia (16), and multiple FIV positive tests regardless of symptoms (15). For write-in answers to this question, 99 organizations explained criteria related to a cat's quality of life or suffering, and 12 cited issues with trauma, pain, or injury.

### Data and Research

Regarding why organizations collect data about feral cats, the most popular answer options selected were applying for new grants and funding (248), internal activity reporting (190), periodically analyzing progress and impact (171), reports to current funders (150), modifying or expanding future trapping efforts (144), public presentations and documents (118), challenging claims made by those who oppose TNR (117), creating maps, graphs, and diagrams (107), campaigns aimed at changing laws (92), collecting data without using it (37), and some write-in answers. By combining categories to better summarize data use trends, the most common uses for data collected by feral cat groups were administrative and fundraising (611), activism, education, and outreach (329), monitoring population impact (320), creating maps, graphs, and diagrams (107), collecting data without using it (37), and medical reasons (6).

We asked respondents which methods they currently used to determine whether their program was effective at saving the lives of cats and/or reducing outdoor cat populations. The most popular answer options selected were feedback from trappers/colony caretakers based on their judgement of cat numbers (268), tracking shelter cat intake (177), tracking shelter kitten intake (155), tracking shelter cat euthanasia (126), monitoring target cat populations at regular intervals to obtain a count or estimate of abundance or density (96), monitoring target cat populations at regular intervals to obtain an estimate of proportion of kittens (79), tracking cat nuisance calls made to animal control (72), monitoring target cat populations at regular intervals to obtain an estimate of sterilization rate (69), and some write-in answers. By combining categories to better summarize types of program efficacy metrics, most were indirect (541) and anecdotal (289), although some were analytical (245).

One means of assessing program impact is tracking the approximate number of cats on the landscape. Our survey asked organizations if they had ever attempted to estimate the number of outdoor cats in a given area, and the most popular response was no (240). In asking what method had been used by those who had attempted to estimate cat numbers, the most popular was asking colony caretakers to count or estimate their cats (174). The most common write-in answer for estimating cat populations referenced using a human-to-cat population ratio (15), a rough guesswork method wherein one divides the human population by some number to get a general idea of how many cats might live in one's service area.

We asked organizations if they had ever reached out to an academic or researcher for assistance with collecting data, analyzing data, or planning any aspect of their program. A vast majority had not (90.2%), and some were unsure (5.6%) or reported yes (4.2%) (*n* = 449). By grouping the write-in answers for those who had sought help, the 2 most common type of entities contacted were veterinarians and academics (6) and cat welfare organizations (5), and the only motivating needs mentioned by more than 1 group were planning their spay/neuter programs (2) and quantifying cats (2). Inversely, we also asked if a group had ever been contacted by an academic or researcher who wanted to work with them or collect data about their organization. While a majority still reported no (82.2%), more contact had been initiated in this direction, with 8.4% unsure and 9.4% reporting yes (*n* = 466). In asking those who reported yes to explain who had contacted them and why, the most common write-in explanations were cat welfare organizations (13), students (9), and academics (7). The write-in reasons for the contact included someone seeking statistics and data (7), interest in animal welfare and behavior (7), bird and wildlife issues (5), disease and medical issues (5), and seeking biological samples (4).

Our survey asked whether respondents would utilize expert assistance in designing and interpreting their data collection if it were available, 14.7% replied no, 29.9% were unsure, 50.9% were interested but only if such assistance is provided without cost, and 4.5% were interested and willing to pay a reasonable fee (*n* = 462).

We proposed 3 areas in which research occurs around feral cats and asked organizations to rate each topic as something they would definitely, possibly, unsure, unlikely, or not be collaborate with researchers to study. Both of the cat-focused options received high support, with 51.6% definitely interested in research aimed at improving the welfare of feral cats (*n* = 467), and 46.5% definitely interested in research aimed at improving the health/welfare of owned cats (*n* = 467). However, for research not geared toward helping cats, support waned. Here, 24.7% were definitely interested in research aimed at studying public health issues (*n* = 466), and 21.5% definitely interested in research aimed at studying cat impacts on birds and wildlife (*n* = 466).

### Bird and Wildlife Issues

We asked if organizations had an official position (such as a statement on their website) about the impact of outdoor cats on birds and wildlife, and if so, which out of 3 options was closest to that position. Most (83.5%) respondents indicated no official position or statement, 8.5% had the position that cats rarely or never have a serious impact on birds or other wildlife, 5.6% had a position that cats may have a serious impact on birds or other wildlife in some places but little or no serious impact in other places, and 2.4% had a position that cats often have a serious impact on birds and/or other wildlife (*n* = 449). We further inquired if organizations had an official position (such as a statement on their website) about how TNR programs change the impact of outdoor cats on birds and wildlife, and if so, which of 4 options was closest to that position. Similarly to above, 75.7% had no official position or statement. Of the rest, 19.9% had a position that TNR programs generally reduce these impacts, 3.1% had a position that TNR programs have impacts that vary from place to place, 5 groups (1.1%) had a position that TNR programs generally do not change these impacts, and a single group (0.2%) had a position that TNR programs generally increase these impacts (*n* = 453).

**Figure 5:**
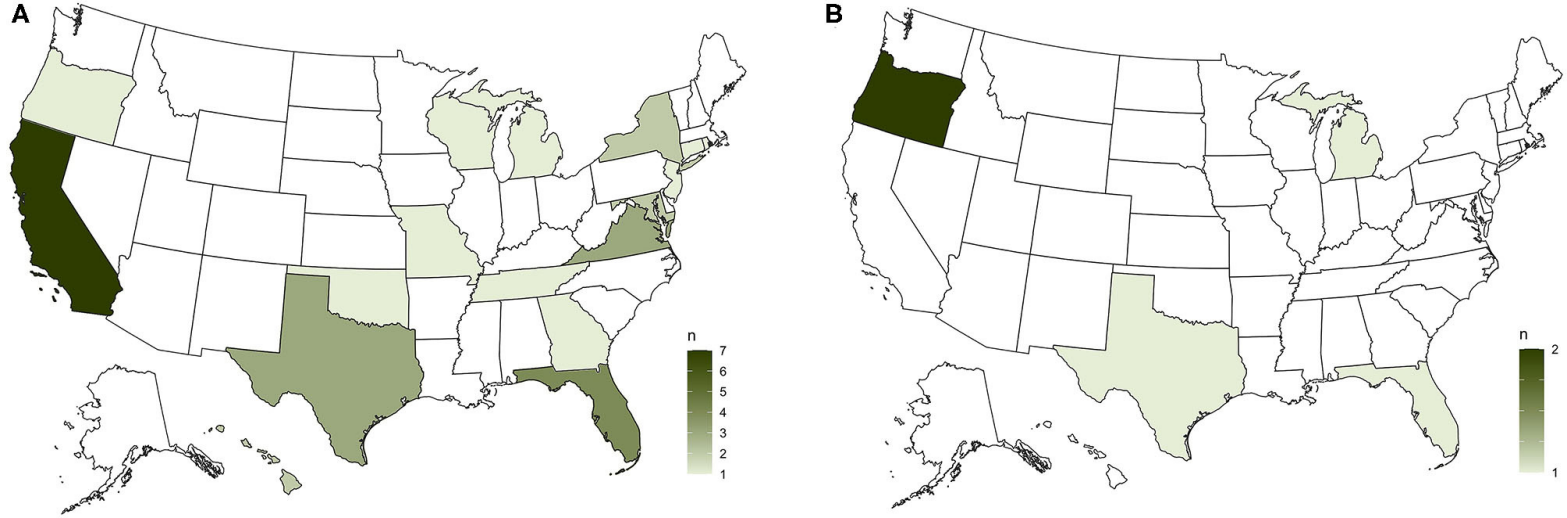
**(A)** Similarly to our question about animal control authorities, public/overt conflict between feral cat advocates and wildlife and bird people was most reported from California. **(B)** Conversely, Oregon was the only state with more than one organization reporting feral cat advocates as engaged in active collaboration and working toward shared goals with wildlife and bird people.

When asked to describe the current relationship between feral cat people and wildlife/bird people in their area, nearly half (42.9%) reported neutral or no interactions, 7.2% reported public/overt conflict, 35.2% reported some tension between groups, 4.8% reported some efforts being made toward bridge-building, 1.1% reported active collaboration and working toward shared goals, and 8.9% reported that they serve many locations and each is different (*n* = 457) (see Figure 5). Finally, to learn more about how positive collaborations occurred, and if it seemed directed formally by organizations or personally by individuals, we asked how that process started. The most commonly-selected answer options were that individuals involved in feral cat issues reached out to individuals they know who were involved in wildlife/bird issues (33), feral cat organizations formally reached out to wildlife/bird organizations (15), working together grew out of tension or public conflict (15), individuals involved in wildlife/bird issues reached out to individuals they know who are involved in feral cat issues (5), and wildlife/bird organizations formally reached out to feral cat organizations (1).

### Best Practice Adherence

By requesting organizations list which guidelines they used in shaping their TNR and medical practices, we identified the 5 most popular resources for investigating best practice adherence (not mutually exclusive): 237 respondents indicated that they used the Guide to Trap-Neuter-Return (TNR) and Colony Care (32), 149 used the Best Friends Community Cat Programs Handbook (online) (33), 148 used the Neighborhood Cats TNR Handbook (34), 137 used Alley Cat Allies Veterinary Resource Center (online) (35) and 90 used the Association of Shelter Veterinarians Guidelines for Spay-Neuter Programs (36). Twelve of our survey questions had an answer or answers supported by a majority or plurality of these guidelines which had a recommendation on the issue. See Table 1 for a summary.

**Table 1:** Twelve questions used in the creation of our best practice adherence model.

**Question**	**Popular advice aligns with survey answer option(s)**
Has your organization consulted with an attorney regarding legal problems that could arise from your work?	Yes, in the past; Yes, on an ongoing basis
Are feral cats scanned for microchips during your TNR process?	Always
For kitten spay/neuter, what is the minimum weight and/or age to determine if kittens can have surgery?	2.0 pounds; 8 weeks/2 months
How does your organization mark feral cats as sterilized? *Ear notches*	Never
How does your organization mark feral cats as sterilized? *Ear tips*	Always
What clinical services do you provide to feral cats? *De-worming/endoparasite treatment*	Discretion of vet/tech; Caretaker request
What clinical services do you provide to feral cats? *Flea/ectoparasite treatment*	Discretion of vet/tech; Caretaker request
What clinical services do you provide to feral cats? *Microchipping*	Discretion of vet/tech; Caretaker request
What clinical services do you provide to feral cats? *Rabies vaccination*	Routine
What clinical services do you provide to feral cats? *FeLV testing*	Not offered
What clinical services do you provide to feral cats? *FIV testing*	Not offered
What clinical services do you provide to feral cats? *Meloxicam or other injectable pain relief*	Routine

We compared models using stepwise backwards model selection by AIC (see Table 2). The best model included whether the organization served urban and suburban areas, whether the organization had a brick-and-mortar facility, whether the organization had its own 501(c)3 federal non-profit status, the US Census Region where an organization is based, and the approximate proportion of animals served annually that were feral cats. Of the answer options for these variables, only 2 were statistically significant using an alpha of 0.05: serving suburban areas and having 501(c)3 status were both predictive of higher best practice scores (term-wise *t-*test *p*-values 0.0243 and 0.0213, respectively) (see Table 3) for ANOVA results.

**Table 2:** A comparison of model terms and their test statistics.

	**Df**	**Deviance**	**AIC**	**ΔAIC**	**LRT**	**Pr(>Chi)**
<none>		669.41	1884.3			
–serve urban	1	671.59	1884.5	0.2	2.175	0.140309
–brick and mortar	2	673.62	1884.5	0.2	4.201	0.122410
+ proportion feral	3	664.53	1885.4	1.1	4.882	0.180649
+ serve rural	1	669.13	1886.0	1.7	0.287	0.592200
−501c3	2	676.19	1887.1	2.8	6.778	0.033744
–serve suburban	1	674.48	1887.3	3.0	5.068	0.024366
+ active volunteers	5	663.05	1887.9	3.6	6.367	0.272096
+ geographical scope	4	665.07	1887.9	3.6	4.349	0.360794
+ number of paid_employees	4	665.55	1888.4	4.1	3.861	0.425090
–census_region	3	681.77	1890.6	6.3	12.360	0.006246
–number of ferals served	8	714.44	1913.3	29.0	45.021	3.646e-7

**Table 3:** ANOVA results for terms within the best model.

**Terms**	**Df**	**Deviance**	**Residual Df**	**Residual Deviance**	**Pr(>Chi)**
census region	3	10.345	561	777.97	0.015848
Serve urban	1	20.690	560	757.28	5.401e-6
Serve suburban	1	11.322	559	745.96	0.000766
501c3	2	20.007	557	725.96	4.523e-5
Brick and mortar	2	11.519	555	714.44	0.003152
Number of ferals served	8	45.021	547	669.41	3.646e-7

## Discussion

### Overview

To the best of our knowledge, this is the largest and most comprehensive study of its type, revealing the most complete available picture of what constitutes the standard practices, opinions, assumptions, and attitudes of organizations serving feral and community cats in the United States. Our large volume of responses from across the country show that a majority of respondent organizations generally appear to face the same challenges, make similar decisions, rank comparable priorities, and offer the same types of care to the feral cats they serve.

There are also minority practices that may be of interest to the animal welfare community. As described in more detail below, we suggest that these findings may in some cases be as notable as majority responses, either in cases where improvement and modernization is warranted or where a small number of groups are leading the way in staking out better solutions.

The following are findings that may be especially interesting or relevant to readers.

### Environmental, Human, and Public Health

Although suspected environmental exposure observations are potentially subjective and largely unconfirmed by a veterinarian or diagnostic testing, they could point to areas where more research is needed. While the survey did not specifically explore whether individuals noting these events then notified public health or other health professionals, the findings indicate that there could be benefit from greater communication between the feral cat welfare community and local public health resources in order to better identify and reduce environmental health risks to both cats and people. This is of additional environmental justice importance considering that half of surveyed organizations report prioritizing trapping cats in low-income areas.

While rabies vaccination is the most common veterinary service apart from sterilization offered by our respondent organizations, it is still not considered routine by all, possibly as a cost-cutting measure. Only a handful reported receiving assistance from government entities in the form of rabies vaccines. Public health and rabies control programs should supply funding for rabies vaccines to feral cat organizations, which benefits the entire community by reducing the number of potential rabies carriers.

### Caretaking, Trapping, and Release

In deciding where to trap cats, a majority of respondents prioritized factors such as intensive and colony-level trapping to get high sterilization coverage, data-driven area selection based on where cats have been entering the shelter system, as well as providing coverage to low-income neighborhoods which tend to lack access to affordable veterinary services. However, there were also 102 organizations that prioritized an even distribution of sterilization services. This latter focus, while perhaps seeming the most fair at face value, is discouraged by experts as a being an inefficient use of time compared to mass trappings (34). Population modeling research has shown that low intensity sterilization is less effective at both reducing preventable cat deaths and decreasing cat population sizes than high intensity sterilization efforts (37).

### Clinical and Medical Issues

Although the prevalence of FIV FeLV has been extensively studied (38), the dispensation of affected feral cats varies and remains largely up to the individual or organization caring for a given animal. While a majority of our respondents reported not routinely testing for these retroviruses, 21.9 and 23.2% did test all cats for FIV and FeLV, respectively. Routine testing of feral cats in TNR programs is not in alignment with advice from professional bodies (39) or advocates (40) on the grounds that doing so is an inefficient use of limited financial resources that could be better spent on sterilization efforts. This recommendation takes resources and capacities for care into account (41), but its adoption is likely dependent on many factors including individual experiences, level of education, cultural acceptance within their communities and access to financial resources. Further, the decision to euthanize should be based on severity of symptoms and quality of life issues, not solely on FIV or FeLV status.

The main purpose of identification microchipping is to reunite lost animals with their owners. Since some cats trapped and presumed to be feral are actually lost pets, the scanning of all cats should be routine in every TNR program. However, only 52.7% of respondents reported always scanning cats for microchips, which highlights the issue of lost reunification opportunities.

A minority of respondents reported using antibiotics not marketed or approved for use in cats, such as aquarium, feed store, or human formulations. This is concerning, as it could be contributing to antibiotic resistance in those communities. It reveals a need for greater access to affordable veterinary care and oversight outside of sterilization and vaccination services, including cases that may warrant antibiotic use.

### Data and Research

For groups that collect data, the most commonly reported motivation for data collection is to meet administrative needs and/or to support fundraising efforts. Only a minority of respondents collected data to assess population level impacts or reported making attempts to engage in more active forms for data exploration, such as mapping or charting.

Most respondents attempt to determine program effectiveness, but a large majority of these do so anecdotally or by relying on indirect measures, such as shelter euthanasia. Less than one-quarter attempt to determine impacts more analytically.

Very few groups have attempted to engage assistance from entities that could provide technical assistance in data collection or analysis, but somewhat more have been contacted by such entities. Slightly over half of respondents would be willing in principle to accept this assistance, but only under certain circumstances. These include the absence of any additional cost, and a perceived motivation by the technical partner for improving cat welfare. Willingness to collaborate with a technical partner fall if the goal of the collaboration involves quantifying cat impacts on wildlife or public health.

Collectively, these responses indicate a TNR constituency that is utilizing data for program support in only a very limited fashion, we infer largely to meet the requirements of funders or to help secure additional funding. Collaborations to improve the use of data in TNR programs are of interest to many TNR practitioners, but willingness to incur costs to secure these services is very limited. There also appears to be substantial discomfort with the idea of investigating wildlife or public health issues during the course of collecting data in conjunction with TNR programs. This suggests a need for continuing outreach and education to make the field more comfortable with the idea of data driven cat population management, and the development of support services to facilitate the use of these tools and integrate them incrementally into routine TNR practice.

### Challenges and Caveats

As with all voluntary response surveys, our respondents might not be entirely representative of our target population. Further, by conducting our survey online through social media, animal welfare websites, and email contact lists, we were unable to make contact with organizations who are not connected to such resources. This could lead to an under-sampling of the most isolated organizations.

Despite our survey being conducted transparently by people with long-term involvement in animal welfare, One Health, and spay/neuter work, there were some accusations that we were “bird people” infiltrating cat welfare Facebook Groups with the intention of spying on cat advocates and harming cats. This may have reduced participation.

Our other key challenge regards whether respondents had the knowledge to answer certain questions. As most organizations reported that they did not operate their own clinics and are presumably reliant on 1 or more third party veterinarians, accurately reporting their veterinary decision-making criteria to us was likely to be difficult. This problem is highlighted by findings such as the 44 organizations which clicked the option indicating that they routinely amputate tails as part of their TNR process. We included rare procedure items in our list to learn whether they were offered to feral cats at all, and we were not expecting so many people to rate it as “routine” rather than “not offered” or “performed at the discretion of a veterinarian.” Despite our explanation that routine means “done to every animal,” we posit these implausible answers about rare procedures may have been interpreted as routinely done to every animal presenting with a need. Further, 28.3% of our respondents reported that they use perioperative antibiotics for routine spay/neuter. If over 1 quarter of feral cat organizations are giving antibiotics to all sterilization patients, this would be a concerning finding. However, since this question used a medical term (“perioperative”) and may have been subject to the same misunderstanding as other questions which contained the word “routine,” we believe this figure skews high. Some of the issues covered in our study would require the collection of medical records to investigate more thoroughly and accurately. The more technical a question, the more we urge caution about some of our results as there is likely a margin of misunderstanding by survey respondents.

This issue arose not only with medical topics, but also with our question on methods of cat population estimation. To the best of our knowledge at the time of this survey, only the Feral Cat Coalition of Oregon was engaged in scientific cat population monitoring through their collaboration with Portland Audubon (42). Yet, 19 organizations clicked a box indicating that they were using mark-recapture population estimation, and 17 indicated they were using transect counts to determine the efficacy of their work. While such findings would be excellent news, we believe it highly unlikely that so many organizations would be conducting rigorous cat population data collection and research programs without those efforts being publicized within the animal welfare community or known privately by the study authors.

We believe that the confusion apparent in some of our questions indicates simple misunderstandings on the part of respondents, rather than a malicious attempt to deceive. Moving forward, our responsibility as researchers is to put more thought into ensuring that the next iteration of this survey will focus on questions that can be understood and answered by anyone at an organization, not just someone with a strong veterinary and scientific background.

## Conclusions

The focus of the present study was not to make recommendations for ideal policies on the matters covered in our survey. There are many veterinary bodies and major animal welfare organizations that publish recommendations for feral cat care and high volume spay/neuter, and we hope these entities can use our results to improve or add emphasis in their materials as they evolve. As we discovered in identifying our set of the most agreed-upon topics to investigate best practice adherence, there were only a dozen issues covered by our survey where the most popular how-to guides were largely in agreement. This demonstrates an area where upper-level interorganizational collaboration and cooperation could result in a more standardized community of practice in the feral cat world.

## Data Availability Statement

The original contributions presented in the study are included in the article/Supplementary Material, further inquiries can be directed to the corresponding author/s.

## Ethics Statement

The studies involving human participants were reviewed and approved by University of Washington Human Subjects Division. The patients/participants provided their written informed consent to participate in this study.

## Author Contributions

SA co-designed the study, developed the questionnaire, solicited participation for the survey, performed the statistical analysis, created figures, and wrote the manuscript. JMB co-designed the study, developed the questionnaire, and contributed to the manuscript. JDB and PR assisted in developing the questionnaire and contributed to the manuscript. All authors contributed to the article and approved the submitted version.

## Funding

This study received no external funding. Its open access article processing fee was paid by SA and JMB.

## Conflict of Interest

The authors declare that the research was conducted in the absence of any commercial or financial relationships that could be construed as a potential conflict of interest.

## Publisher's Note

All claims expressed in this article are solely those of the authors and do not necessarily represent those of their affiliated organizations, or those of the publisher, the editors and the reviewers. Any product that may be evaluated in this article, or claim that may be made by its manufacturer, is not guaranteed or endorsed by the publisher.
