# Corrigendum: “State of the Mewnion”: Practices of Feral Cat Care and Advocacy Organizations in the United States

**DOI:** 10.3389/fvets.2021.846256

**Published:** 2022-01-25

**Authors:** Sabrina Aeluro, Jennifer M. Buchanan, John D. Boone, Peter M. Rabinowitz

**Affiliations:** ^1^Kitizen Science, Seattle, WA, United States; ^2^Feral Cat Spay/Neuter Project, Lynnwood, WA, United States; ^3^Great Basin Bird Observatory, Reno, NV, United States; ^4^Center for One Health Research, Department of Environmental & Occupational Health Sciences, School of Public Health, University of Washington, Seattle, WA, United States

**Keywords:** free-roaming cat, feral cat, community cat, spay/neuter, trap neuter return, shelter neuter return, return to field, TNR

In the original article, the **Abstract** contained a results error. It should state “1 or 2 nights for females.” The corrected paragraph appears below.

Over the last several decades, feral cats have moved from the fringes to the mainstream in animal welfare and sheltering. Although many best practice guidelines have been published by national non-profits and veterinary bodies, little is known about how groups “in the trenches” actually operate. Our study sought to address that gap through an online survey of feral cat care and advocacy organizations based in the United States. Advertised as “The State of the Mewnion,” its topics included a range of issues spanning non-profit administration, public health, caretaking and trapping, adoptions of friendly kittens and cats, veterinary medical procedures and policies, data collection and program efficacy metrics, research engagement and interest, and relationships with wildlife advocates and animal control agencies. Respondents from 567 organizations participated, making this the largest and most comprehensive study on this topic to date. Respondents came primarily from grassroots organizations. A majority reported no paid employees (74.6%), served 499 or fewer feral cats per year (75.0%), engaged between 1 and 9 active volunteers (54.9%), and did not operate a brick and mortar facility (63.7%). Some of our findings demonstrate a shared community of practice, including the common use of a minimum weight of 2.0 pounds for spay/neuter eligibility, left side ear tip removals to indicate sterilization, recovery holding times after surgery commonly reported as 1 night for male cats and 1 or 2 nights for females, requiring or recommending to adopters of socialized kittens/cats that they be kept indoor-only, and less than a quarter still engaging in routine testing of cats for FIV and FeLV. Our survey also reveals areas for improvement, such as most organizations lacking a declared goal with a measurable value and a time frame, only sometimes scanning cats for microchips, and about a third not using a standardized injection site for vaccines. This study paints the clearest picture yet available of what constitutes the standard practices of organizations serving feral and community cats in the United States.

The authors apologize for this error and state that this does not change the scientific conclusions of the article in any way. The original article has been updated.

## Publisher's Note

All claims expressed in this article are solely those of the authors and do not necessarily represent those of their affiliated organizations, or those of the publisher, the editors and the reviewers. Any product that may be evaluated in this article, or claim that may be made by its manufacturer, is not guaranteed or endorsed by the publisher.

