# Supplementary material for: “State of the Mewnion”: Practices of Feral Cat Care and Advocacy Organizations in the United States
Source: Front Vet Sci. 2021 Dec 14;8:791134. doi: 10.3389/fvets.2021.791134 (PMC8712445; doi:10.3389/fvets.2021.791134)
Supplement: Supplementary file 1 [file Table_1.DOCX]

**State of the Mewnion Survey 2018 – Complete Results with Unrounded Percentages**

**Organization Basics**

Where is your organization located/based? (Percentage: all survey respondents.)

Alabama 4 - 0.7054674%

Arizona 9 - 1.5873016%

Arkansas 7 - 1.2345679%

California 55 - 9.7001764%

Colorado 12 - 2.1164021%

Connecticut 11 - 1.9400353%

Delaware 2 - 0.3527337%

District of Columbia 4 - 0.7054674%

Florida 39 - 6.8783069%

Georgia 11 - 1.9400353%

Hawaii 5 - 0.8818342%

Idaho 5 - 0.8818342%

Illinois 19 - 3.3509700%

Indiana 12 - 2.1164021%

Iowa 4 - 0.7054674%

Kansas 8 - 1.4109347%

Kentucky 4 - 0.7054674%

Louisiana 13 - 2.2927690%

Maine 2 - 0.3527337%

Maryland 14 - 2.4691358%

Massachusetts 18 - 3.1746032%

Michigan 18 - 3.1746032%

Minnesota 15 - 2.6455026%

Mississippi 1 - 0.1763668%

Missouri 9 - 1.5873016%

Montana 3 - 0.5291005%

Nebraska 4 - 0.7054674%

Nevada 5 - 0.8818342%

New Hampshire 5 - 0.8818342%

New Jersey 17 - 2.9982363%

New Mexico 4 - 0.7054674%

New York 33 - 5.8201058%

North Carolina 9 - 1.5873016%

North Dakota 1 - 0.1763668%

Ohio 11 - 1.9400353%

Oklahoma 7 - 1.2345679%

Oregon 11 - 1.9400353%

Pennsylvania 25 - 4.4091711%

Rhode Island 2 - 0.3527337%

South Carolina 11 - 1.9400353%

South Dakota 1 - 0.1763668%

Tennessee 10 - 1.7636684%

Texas 38 - 6.7019400%

Utah 5 - 0.8818342%

Virginia 21 - 3.7037037%

Washington 23 - 4.0564374%

West Virginia 5 - 0.8818342%

Wisconsin 15 - 2.6455026%

What is the geographical area that your organization serves most of the time? (Percentage: all survey respondents.)

Neighborhood/development 68 - 11.9929453%

City/town 412 - 72.6631393%

Statewide 57 - 10.0529101%

Multi-state regional 28 - 4.9382716%

National 2 - 0.3527337%

Which areas does your organization serve? Check all that apply. (Percentage: all survey respondents.)

Urban 386 - 68.0776%

Suburban 438 - 77.24868%

Rural 401 - 70.7231%

What is the approximate proportion of animals that your organization serves/advocates for which are feral/community cats? (Including kittens that are the offspring of such cats.) (Percentage: all survey respondents.)

¼ or less 104 - 18.34215%

¼ to ½ 117 - 20.63492%

½ to ¾ 94 - 16.57848%

¾ or more 52 - 44.44444%

Does your organization have its own 501(c)3 status? (Percentage: all survey respondents.)

Yes 418 - 73.721340%

No 118 - 20.811287%

No, but we can receive tax deductible donations through another organization 31 - 5.467372%

Does your group have a brick and mortar facility? (Apart from operating out of a person’s home. (Percentage: all survey respondents.)

Yes 176 - 31.040564%

Yes, as a part of a shared space with another project or within a larger organization 30 - 5.291005%

No 361 - 63.668430%

If you provide services or caretaking, what is the approximate number of feral/community cats that you serve per year? (Percentage: all survey respondents.)

Fewer than 99 202 - 35.6261023%

100-499 223 - 39.3298060%

500-999 54 - 9.5238095%

1000-2499 51 - 8.9947090%

2499-4999 23 - 4.0564374%

5000-9999 8 - 1.4109347%

10000-14999 2 - 0.3527337%

15000-19999 3 - 0.5291005%

20000 or more 1 - 0.1763668%

Is your group a project/club run by veterinary students? (Percentage: all survey respondents.)

Yes 4 - 0.7054674%

No 563 - 99.2945326%

What is the number of paid employees in your group? (Or, if your organization does some feral/community work alongside other functions, what is the number of paid employees just within your feral/community cat section?) (Percentage: all survey respondents.)

0 423 - 74.603175%

1-4 77 - 13.580247%

5-9 28 - 4.938272%

10-19 21 - 3.703704%

20 or more 18 - 3.174603%

What is the number of active volunteers in your group? (Or, if your organization does some feral/community work alongside other functions, what is the number of volunteers just within your feral/community cat section?) (Percentage: all survey respondents.)

0 20 - 3.527337%

1-9 311 - 54.850088%

10-19 97 - 17.107584%

20-49 73 - 12.874780%

50-99 24 - 4.232804%

100 or more 42 - 7.407407%

What does your organization do? Categorize each as a primary, secondary, or not offered. (Percentage: all survey respondents, percent is for each item.)

Direct feeding and colony care for feral/community cats

Not offered 195 - 34.39153% Primary 154 - 27.16049% Secondary 218 - 38.44797%

Direct trapping of feral/community cats for TNR

Not offered 81 - 14.28571% Primary 304 - 53.61552% Secondary 182 - 32.09877%

Coordinating volunteers who are trapping feral/community cats for TNR

Not offered 152 - 26.80776% Primary 171 - 30.15873% Secondary 244 - 43.03351%

Training or holding workshops for caretakers and trappers

Not offered 318 - 56.08466% Primary 68 - 11.99295% Secondary 181 - 31.92240%

We operate our own clinic focused on feral/community cat care

Not offered 465 - 82.010582% Primary 48 - 8.465608% Secondary 54 - 9.523810%

FREE sterilization/vaccination/basic medical care for feral/community cats

Not offered 285 - 50.26455% Primary 160 - 28.21869% Secondary 122 - 21.51675%

LOW-COST sterilization/vaccination/basic medical care for feral/community cats

Not offered 243 - 42.85714% Primary 180 - 31.74603% Secondary 144 - 25.39683%

FREE sterilization/vaccination/basic medical care for owned cats from low-income households

Not offered 356 - 62.78660% Primary 78 - 13.75661% Secondary 133 - 23.45679%

LOW-COST sterilization/vaccination/basic medical care for owned cats from low-income households

Not offered 289 - 50.97002% Primary 115 - 20.28219% Secondary 163 - 28.74780%

FREE sterilization/vaccination/basic medical care for cats from animal shelters/rescues

Not offered 459 - 80.95238% Primary 54 - 9.52381% Secondary 54 - 9.52381 %

LOW-COST sterilization/vaccination/basic medical care for cats from animal shelters/rescues

Not offered 407 - 71.78131% Primary 82 - 14.46208% Secondary 78 - 13.75661%

Socializing/fostering adult feral/community cats for adoption

Not offered 219 - 38.62434% Primary 114 - 20.10582% Secondary 234 - 41.26984%

Socializing/fostering kittens from feral/community cats for adoption

Not offered 109 - 19.22399% Primary 219 - 38.62434% Secondary 239 - 42.15168%

Running a sanctuary for feral/community cats

Not offered 411 - 72.48677% Primary 70 - 12.34568% Secondary 86 - 15.16755%

We are an animal shelter with a return-to-field program for feral/community cats

Not offered 397 - 70.01764% Primary 95 - 16.75485% Secondary 75 - 13.22751%

Operating a “working cats” program for urban/suburban mousers

Not offered 371 - 65.432099% Primary 53 - 9.347443% Secondary 143 - 25.220459%

Operating a “barn cats” program for rural mousers

Not offered 293 - 51.67549% Primary 80 - 14.10935% Secondary 194 - 34.21517%

Providing disaster relief for feral/community cats as needed

Not offered 396 - 69.841270% Primary 26 - 4.585538% Secondary 145 - 25.573192%

Engaging in organization-level training and mentorship to other feral/community cat groups

Not offered 376 - 66.313933% Primary 37 - 6.525573% Secondary 154 - 27.160494%

Public education and awareness raising around feral/community cat welfare issues

Not offered 114 - 20.10582% Primary 159 - 28.04233% Secondary 294 - 51.85185%

Resource sharing/community building for those already working to help feral/community cats

Not offered 160 - 28.21869% Primary 132 - 23.28042% Secondary 275 - 48.50088%

Campaigning for law and policy changes around feral/community cats

Not offered 295 - 52.02822% Primary 66 - 11.64021% Secondary 206 - 36.33157%

Coalition-building between feral/community cat advocates and bird/conservation advocates

Not offered 459 - 80.952381% Primary 26 - 04.585538% Secondary 82 - 14.462081%

Providing grants and funding organizations doing feral/community cat work

Not offered 516 - 91.005291% Primary 15 - 02.645503% Secondary 36 - 6.349206%

Training of volunteers and caregivers regarding trapping, colony care, and best practices

Not offered 172 - 30.33510% Primary 145 - 25.57319% Secondary 250 - 44.09171%

Participating in research projects

Not offered 474 - 83.5978836% Primary 5 - 0.8818342% Secondary 88 - 15.5202822%

**Administration and Policy**

Does your organization currently have at least one declared goal that includes both a measurable value and a timeframe? Such as, “reduce the outdoor cat population of our town 25% by 2025” or “provide 1000 free spay/neuter surgeries every year.” (Percentage: respondents to this question.)

Yes 181 - 32.1492%

No 382 - 67.8508%

Is TNR explicitly allowed or endorsed by local laws and animal control ordinances where you operate? (Percentage: respondents to this question.)

Yes 263 - 46.466431%

No 101 - 17.844523%

Unsure 56 - 9.893993%

It varies, we operate across many jurisdictions 146 - 25.795053%

If TNR is not explicitly legal in your area, are there local laws that could be used, or are actively being enforced, to prohibit or limit feral/community cat care, feeding, or TNR? Rate each item. (Percentage: respondents to question, percent is for each item.)

Laws against feeding

Could Be Used 161 - 39.26829% Actively Enforced 43 - 10.48780% Unknown 206 - 50.24390%

Laws defining outdoor cat feeders as the cat’s owner

Could Be Used 164 -.39.80583% Actively Enforced 63 - 15.29126% Unknown 185 - 44.90291%

Pet licensing laws

Could Be Used 158 - 38.53659% Actively Enforced 73 - 17.80488% Unknown 179 - 43.65854%

Mandatory “stray” holding periods

Could Be Used 105 - 25.54745% Actively Enforced 171 - 41.60584% Unknown 135 - 32.84672%

Pet limits

Could Be Used 174 - 41.82692% Actively Enforced 103 - 24.75962% Unknown 139 - 33.41346%

Animal control of “nuisance” animals

Could Be Used 185 - 44.36451% Actively Enforced 116- 27.81775% Unknown 116 - 27.81775%

Required colony registration

Could Be Used 88 - 21.782178% Actively Enforced 16 - 3.960396% Unknown 300 - 74.257426%

Mandatory spay/neuter requirements

Could Be Used 115 - 28.822055% Actively Enforced 33 - 8.270677% Unknown 251 - 62.907268%

Microchipping requirements

Could Be Used 103 - 25.814536% Actively Enforced 20 - 5.012531% Unknown 276 - 69.172932%

Leash laws which include cats

Could Be Used 121 - 29.729730% Actively Enforced 26 - 6.388206% Unknown 260 - 63.882064%

Abandonment laws

Could Be Used 204 - 49.39467% Actively Enforced 57 - 13.80145% Unknown 152 - 36.80387%

Laws restricting veterinarians’ abilities to provide free/low-cost services

Could Be Used 47 - 12.020460% Actively Enforced 7 - 1.790281% Unknown 337 - 86.189258%

Other: Write-in answers grouped/summarized by authors. (Other percentages: all survey respondents)

Trapping prohibitions 4 - 0.7054674%

Legal issues with TNR in Los Angeles 2 - 0.3527337%

Trespassing and property crimes 2 - 0.3527337%

Outdoor cats 2 - 0.3527337%

Theft 1 - 0.1763668%

Quarantine 1 - 0.1763668%

Incorrect information given out by law enforcement about TNR being illegal 1 - 0.1763668%

Vaccination requirements 1 - 0.1763668%

Has your organization consulted with an attorney regarding legal problems that could arise from your work? (Percentage: respondents to this question.)

Yes, at some point in the past 137 - 24.551971%

Yes, on an ongoing basis 46 - 8.243728%

No 341 - 61.111111%

Unsure 34 - 6.093190%

How would the current relationship between feral/community cat advocates and animal control authorities in your area best be described? (Percentage: respondents to this question.)

Public/overt conflict occurring 22 - 3.907638%

Some tension between groups 62 - 11.012433%

Neutral or no interactions 95 - 16.873890%

Some efforts being made towards bridge-building 98 - 17.406750%

Active collaboration and working towards shared goals 185 - 32.859680%

We serve many locations, each is different 88 - 15.630551%

We are the animal control authorities in our area 13 - 2.309059%

If feral/community cats is one part of what your organization does, are there reasons why you don't serve more feral/community cats? Check all that apply. (Percentage: respondents for each item.)

We are a comprehensive animal program that fulfills many roles 197 - 34.74427

We would do more with feral/community cats if we had specific grants/funding 330 - 58.20106

We don’t have the proper facilities or equipment 182 - 32.09877

Our staff doesn’t have the proper training 31 - 5.467372

Concern about possible injuries to staff and volunteers 15 - 2.645503

There is an alternative for feral/community cat care in our area 53 - 9.347443

Our organization has a policy that prevents (more) care of feral/community cats 7 - 1.234568

Unsure 6 - 1.058201

Other: Write-in answers grouped/summarized by authors. (Other percentages: all survey respondents)

Need for volunteers 74 - 13.05115

Need for personnel/staff 32 - 5.643739

Need for SN services 26 - 4.585538

Small or rural group 24 - 4.232804

Need for trappers and places to trap 23 - 4.056437

Need for transportation 8 - 1.410935

Need for foster homes 8 - 1.410935

Limits of partner organization(s) 8 - 1.410935

Social/community acceptance 7 - 1.234568

Laws 5 - 0.8818342

Good news (cats under control) 4 - 0.7054674

Need for better organization/planning 3 - 0.5291005

Physical ability 2 - 0.3527337

People sometimes discuss “feral cats,” “stray cats,” “barn cats,” or “community cats” as separate categories, using their own definitions of these groups. Does your organization have policies or priorities that differentiate between classifications of outdoor, free-roaming cats? (Percentage: respondents to this question.)

Yes 207 - 36.637168%

No 344 - 60.884956%

Unsure 14 - 2.477876%

What resources (books, websites, blogs, Facebook groups, etc.) does your organization regularly use and trust for updates, information, and news about feral/community cat issues? (Sources mentioned by at least three respondents. Resources only mentioned once or twice not tallied here. Non-specific answers such as "Facebook," "Instagram," or "websites" not tallied.)

Alley Cat Allies 223

Best Friends 56

Neighborhood Cats 46

HSUS 44

ASPCA 33

Community Cats Podcast 21

Maddies Fund 17

Vox Felina / Peter Wolf 10

Million Cat Challenge 9

Community Cats United 8

PetSmart 6

Julie Levy/University of Florida 6

Operation Catnip 5

Animal Sheltering Magazine 5

Alley Cat Rescue 5

San Antonio FCC 4

UC Davis 4

Community Cat Coalition 4

Humane Alliance 4

Alley Cat Advocates 3

Stray Cat Alliance 3

Austin Pets Alive 3

Kitten Lady 3

FCCC 3

Petfinder 3

Peggy Adams Animal Rescue League 3

Does your organization have a locally-focused online discussion group or email list where feral/community cat advocates can ask questions, share resources, seek assistance, and support one another? (Percentage: respondents to this question.)

Yes 210 - 37.10247%

No 356 - 62.89753%

**Environmental, Human, and Public Health**

Are you seeing health issues in feral/community cats that are suspected as being caused by exposures to toxins or environmental contaminants? (Percentage: respondents to this question.)

No 321 - 59.00735%

Unknown 148 - 27.20588%

Yes 75 - 13.78676%

Optional write in explanation: answers categorized by authors. (Percentages: of all survey respondents)

Chemical/toxic exposure 51 - 8.994709%

Infectious disease 10 - 1.763668%

Climate/weather 5 - 0.8818342%

Reproductive/birth defects 4 - 0.7054674%

Suspected cancer/carcinogens 4 - 0.7054674%

Firearms 3 - 0.5291005%

Other 14 - 2.469136%

Do you have a formal process for staff or volunteers who receive bites or other injuries from feral/community cats? (Percentage: respondents to this question.)

Yes 217 - 39.96317%

No 326 - 60.03683%

Does your organization maintain insurance for staff and volunteers to cover medical care for injuries sustained during work with feral/community cats? (Percentage: respondents to this question.)

Yes 182 - 33.57934%

No 360 - 66.42066%

Does your organization provide staff and/or volunteers with mental health care resources, such as information on compassion fatigue, support groups for animal welfare workers, suicide and crisis hotlines, or referrals to mental health providers? (Percentage: respondents to this question.)

Yes 70 - 12.89134%

No 473 - 87.10866%

**Caretaking, Trapping, and Release**

Does your group provide feeders/caretakers with any official recommendations or training for best practices, such as the amount of food to provide, information on making outdoor cat shelters, monitoring cat health, dealing with conflicts with neighbors, or how to trap cats? (Percentage: respondents to this question.)

Yes 376 - 72.30769%

No 144 - 27.69231%

Are colonies or colony caretakers in your service area required by law to be registered in some way? (Percentage: respondents to this question.)

Yes 48 - 9.17782%

No 402 - 76.86424%

Unsure 73 - 13.95793%

Regardless of whether registration is required by law, approximately what proportion of colonies or colony caretakers in your service area do you estimate are actually registered? (Percentage: respondents to this question.)

¼ or less 417 - 86.875%

¼ to ½ 32 - 6.666667%

½ to ¾ 8 - 1.666667%

¾ or more 23 - 4.791667%

If you do register colonies or caretakers, where is this information stored? (Percentage: respondents to this question.)

With a government office 26 - 6.878307%

With a private nonprofit/individual 109 - 28.835979%

Both government and private 26 - 6.878307%

Unsure 217 - 57.407407%

If you do not always register colonies or caretakers, why not? Check all that apply. (Percentage: all survey respondents. Will not sum to 100% since not everyone answered and some chose multiple answers.)

Some caretakers have refused 81 - 14.28571%

We believe caretakers might be resistant 78 - 13.75661%

Feeding/TNR is illegal in our area 52 - 9.171076%

We were advised by an attorney to not document colonies/caretakers 11 - 1.940035%

We lack the tools or technical resources to maintain a registry 87 - 15.34392%

We lack the time or personnel to maintain a registry 157 - 27.68959%

We don’t see a reason to register colonies/caretakers 145 - 25.57319%

We are intending on implementing a registry (or better registry) soon 26 - 4.585538%

Other: Write-in answers grouped/summarized by authors. (Other percentages: all survey respondents)

Group has their own records 35 - 6.17284%

Registration is not required 32 - 5.643739%

Fear of how the data could be used 29 - 5.114638%

No way to register 9 - 1.587302%

Another entity has a registry 6 - 1.058201%

For groups trapping feral/community cats for sterilization, how does your organization decide where to trap? Rate each reason as higher priority, lower priority, or not a factor used when trapping. (Percentage: respondents to question, percent is for each item.)

Complaints from the public about number of cats in a location

Higher 335 - 71.42857% Lower 67 - 14.28571% Not a Factor 67 - 14.28571%

Requests from colony caretakers

Higher 371 - 78.601695% Lower 54 - 11.440678% Not a Factor 47 - 9.957627%

Places located conveniently for trappers (such as near their homes)

Higher 151 - 32.33405% Lower 132 - 28.26552% Not a Factor 184 - 39.40043%

Locations that are safe for trappers to work

Higher 174 - 37.17949% Lower 135 - 28.84615% Not a Factor 159 - 33.97436%

Evenly distributing efforts to provide some sterilizations to as many caretakers as possible

Higher 102 - 21.88841% Lower 123 - 26.39485% Not a Factor 241 - 51.71674%

Concentrating efforts in smaller areas to get high sterilization coverage of some areas

Higher 188 - 40.43011% Lower 116 - 24.94624% Not a Factor 161 - 34.62366%

Trapping in one area or colony until all cats are caught and sterilized

Higher 364 - 77.446809% Lower 62 - 13.191489% Not a Factor 44 - 9.361702%

Providing TNR services to low-income neighborhoods

Higher 238 - 50.85470% Lower 102 - 21.79487% Not a Factor 128 - 27.35043%

Locations from which many cats are entering the shelter/animal control system

Higher 188 - 40.43011% Lower 71 - 15.26882% Not a Factor 206 - 44.30108%

Areas where cats are suspected to pose a risk to birds and wildlife

Higher 40 – 8.56531% Lower 84 - 17.98715% Not a Factor 343 - 73.44754%

Based on funding/grants that specify where we will provide services

Higher 136 - 29.05983% Lower 72 - 15.38462% Not a Factor 260 - 55.55556%

Trapping for TNR and relocation to protect cats at risk of harm

Higher 264 - 56.28998% Lower 84 - 17.91045% Not a Factor 121 - 25.79957%

Other

Higher 26 - 18.18182% Not a Factor 117 - 81.81818%

For kittens (born to feral/community cats) under 2 months of age, do you remove them from the outdoors for fostering, socialization, and adoption? (Percentage: respondents to this question.)

Always 247 - 48.431373%

Usually 143 - 28.039216%

Sometimes 81 - 15.882353%

Rarely 30 - 5.882353%

Never 9 - 1.764706%

For kittens (born to feral/community cats) between 2 and 3 months of age, do you remove them from the outdoors for fostering, socialization, and adoption? (Percentage: respondents to this question.)

Always 120 - 23.529412%

Usually 63 - 12.352941%

Sometimes 158 - 30.980392%

Rarely 152 - 29.803922%

Never 17 - 3.333333%

If you do adoptions of socialized feral/community cats or kittens born to feral/community cats, does your group’s adoption information have a position on whether they should be kept as indoor-only animals? (This is regarding adoptions of socialized cats/kittens as traditional pets, not barn cat/working cat programs.) (Percentage: respondents to this question.)

We require that adopted cats/kittens be indoor-only 259 - 52.749491%

We recommend that adopted cats/kittens be indoor-only 140 - 28.513238%

We require that adopted cats/kittens be allowed both indoors and outdoors 0

We recommend that adopted cats/kittens be allowed both indoors and outdoors 6 - 1.221996%

We have no position on where adopters keep their cats/kittens 48 - 9.775967%

Other 38 - 7.739308%

After a routine neuter with no complications, what is your policy on what happens to male cats? (Percentage: respondents to this question.)

Always released on the same day as surgery 34 - 6.666667%

Held overnight and then released 377 - 73.921569%

Held for 2 nights and then released 47 - 9.215686%

Held for 3 nights and then released 15 - 2.941176%

Other 37 - 7.254902%

After a routine spay with no complications, what is your policy on what happens to female cats? (Percentage: respondents to this question.)

Always released on the same day as surgery 14 - 2.723735%

Held overnight and then released 246 - 47.859922%

Held for 2 nights and then released 124 - 24.124514%

Held for 3 nights and then released 67 - 13.035019%

Other 63 - 12.256809%

Does your organization routinely recommend or use any supplements or alternative medicine products with feral/community cats, and if so, which type? Check all that apply (Percentage: all survey respondents. Will not sum to 100% since not everyone answered and some chose multiple answers.)

No supplements/alternative medicine products 323 - 56.96649%

Probiotics (such as FortiFlora™) 97 - 17.10758%

Feliway® 82 - 14.46208%

Rescue Remedy® 52 - 9.171076%

Homeopathic products 38 - 6.70194%

Herbal products 24 - 4.232804%

Glucosamine 18 - 3.174603%

**Clinical and Medical Issues**

What, if any, published guidelines do you use in shaping your own TNR and medical practices? Check all that apply. (Percentage: all survey respondents. Will not sum to 100% since not everyone answered and some chose multiple answers.)

Alley Cat Allies/ASPCA/Mayor’s Alliance for NYC’s Animals Guide to Trap-Neuter-Return 237 - 41.79894%

Best Friends Community Cat Programs Handbook 149 - 26.27866%

Neighborhood Cats TNR Handbook 148 - 26.10229%

Alley Cat Allies Veterinary Resource Center 137 - 24.16226%

Association of Shelter Veterinarians Guidelines for Spay-Neuter Programs 90 - 15.87302%

Community TNR: Tactics and Tools by Bryan Kortis 82 - 14.46208%

ASPCA’s Special Considerations for Community Cats at Spay/Neuter Clinics 79 - 13.93298%

American Association of Feline Practitioners Practice Guidelines 55 - 9.700176%

ISFM Guidelines on Population Management and Welfare of Unowned Domestic Cats 5 - 0.8818342%

ICAM Coalition Humane Cat Population Management Guidance 5 - 0.8818342%

Other: Write-in answers grouped/summarized by authors. (Other percentages: all survey respondents)

Other 18 - 3.174603%

Humane Alliance 4 - 0.7054673%

UF / Operation Catnip 3 - 0.5291005%

UC Davis 2 - 0.3527337%

If your group traps cats but does not operate a clinic, approximately how far must animals be transported to reach your nearest provider of sterilization services for feral/community cats? (Percentage: respondents to this question.)

Under 30 minutes by car 220 - 54.455446%

30-60 minutes by car 157 - 38.861386%

60-90 minutes by car 19 - 4.702970%

90-120 minutes by car 4 - 0.990099%

2-4 hours by car 4 - 0.990099%

4-6 hours by car 0

6 or more hours by car 0

Whether or not you operate your own clinic, approximately how far away is the next-nearest provider of sterilization services for feral/community cats? (Percentage: respondents to this question.)

Under 30 minutes by car 215 - 46.436285%

30-60 minutes by car 192 - 41.468683%

60-90 minutes by car 36 - 7.775378%

90-120 minutes by car 13 - 2.807775%

2-4 hours by car 7 - 1.511879%

4-6 hours by car 0

6 or more hours by car 0

Unknown 0

If you provide or facilitate sterilization and veterinary services, are your fees different for cats reported as owned versus cats reported as being feral/community cats? (Percentage: respondents to this question.)

Yes 185 - 56.40244%

No 143 - 43.59756%

If you provide free or discounted services to low-income caretakers and trappers of feral/community cats, do you have a stated cut-off for what qualifies as “low-income”? (Percentage: respondents to this question.)

Yes 29 - 11.55378%

Decided on a case-by-case basis 61 - 24.30279%

No 161 - 64.14343%

If you answered “yes” above, do you require documentation of low-income status, such as with a pay stub or tax return, or a person’s qualification for federal assistance programs like Medicare? (Percentage: respondents to this question.)

Yes 48 - 15.43408%

No 263 - 84.56592%

If your organization has a standard required fee or suggested donation to pay for veterinary services for feral/community cats, what is that amount in US dollars and what does it include? (Some respondents entered $0.00; zeros were dropped from calculations since this question is about fees.)

Mean Std Dev

For routine female spay $44.57714 22.63081

For routine male neuter $37.71676 18.89092

For routine female spay, plus rabies vaccine $48.06283 24.54204

For routine male neuter, plus rabies vaccine $42.23684 20.60569

For routine female spay, plus rabies and FVRCP vaccines $53.83333 29.24372

For routine male neuter, plus rabies and FVRCP vaccines $48.73316 25.38152

Are feral/community cats scanned for microchips during your TNR process? (Percentage: respondents to this question.)

Always 231 - 52.61959%

Sometimes 152 - 34.62415%

Never 56 - 12.75626%

If you microchip feral/community cats, are the microchips registered somewhere? Check all that apply. (Percentage: all survey respondents. Will not sum to 100% since not everyone answered and some chose multiple answers.)

Yes, registered with a standard pet microchip company’s database 114 - 20.10582%

Yes, registered with a rescue group 73 - 12.87478%

Yes, registered with local animal control 29 - 5.114638%

No, the chip numbers are just for the caretaker’s records 28 - 4.938272%

Other: Write-in answers grouped/summarized by authors. (Other percentages: all survey respondents)

Yes, kept in the clinic records 4 - 0.7054674%

Yes, registered on FoundAnimals 3 - 0.5291005%

What clinical services do you provide to feral/community cats? Categorize each as routine (done to every animal), done at the discretion of a veterinarian or vet tech, provided if requested by a caretaker, or not offered. (Percentage: respondents to question, percent is for each item.)

Flea/ectoparasite treatment

Routine 215 - 50.469484%

Discretion of tech/vet 97 - 22.769953%

Caretaker request 73 - 17.136150%

Not offered 41 - 9.624413%

De-worming/endoparasite treatment

Routine 171 - 40.81146%

Discretion of tech/vet 99 - 23.62768%

Caretaker request 100 - 23.86635%

Not offered 49 - 11.69451%

Rabies vaccination

Routine 383 - 89.485981%

Discretion of tech/vet 7 - 01.635514%

Caretaker request 19 - 4.439252%

Not offered 19 - 4.439252%

FVRCP vaccination

Routine 245 - 59.03614%

Discretion of tech/vet 13 - 3.13253%

Caretaker request 104 - 25.06024%

Not offered 53 - 12.77108%

FeLV vaccination

Routine 43 - 10.886076%

Discretion of tech/vet 13 - 3.291139%

Caretaker request 117 - 29.620253%

Not offered 222 - 56.202532%

Microchipping

Routine 77 - 19.106700%

Discretion of tech/vet 14 - 3.473945%

Caretaker request 153 - 37.965261%

Not offered 159 - 39.454094%

Meloxicam or other injectable pain relief

Routine 185 - 44.68599%

Discretion of tech/vet 135 - 32.60870%

Caretaker request 34 - 8.21256%

Not offered 60 - 14.49275%

Fluids

Routine 46 - 11.302211%

Discretion of tech/vet 261 - 64.127764%

Caretaker request 16 - 3.931204%

Not offered 84 - 20.638821%

FIV testing

Routine 89 - 21.86732%

Discretion of tech/vet 59 - 14.49631%

Caretaker request 163 - 40.04914%

Not offered 96 - 23.58722%

FeLV testing

Routine 95 - 23.17073%

Discretion of tech/vet 62 - 15.12195%

Caretaker request 162 - 39.51220%

Not offered 91 - 22.19512%

Cleaning of wounds/abscesses

Routine 239 - 56.501182%

Discretion of tech/vet 152 - 35.933806%

Caretaker request 15 - 3.546099%

Not offered 17 - 4.018913%

Extraction of diseased teeth

Routine 78 - 18.840580%

Discretion of tech/vet 187 - 45.169082%

Caretaker request 35 - 8.454106%

Not offered 114 - 27.536232%

Dental cleanings

Routine 13 - 3.201970%

Discretion of tech/vet 103 - 25.369458%

Caretaker request 40 - 9.852217%

Not offered 250 - 61.576355%

Enucleation

Routine 42 - 10.370370%

Discretion of tech/vet 208 - 51.358025%

Caretaker request 26 - 6.419753%

Not offered 129 - 31.851852%

Entropion repair

Routine 30 - 7.462687%

Discretion of tech/vet 201 - 50.000%

Caretaker request 23 - 5.721393%

Not offered 148 - 36.815920%

Tail amputation

Routine 44 - 10.653753%

Discretion of tech/vet 256 - 61.985472%

Caretaker request 24 - 5.811138%

Not offered 89 - 21.549637%

Limb amputation

Routine 36 - 8.801956%

Discretion of tech/vet 224 - 54.767726%

Caretaker request 24 - 5.867971%

Not offered 125 - 30.562347%

Declaw of embedded claw

Routine 41 - 10.049020%

Discretion of tech/vet 229 - 56.127451%

Caretaker request 18 - 4.411765%

Not offered 120 - 29.411765%

Umbilical hernia repairs

Routine 106 - 25.853659%

Discretion of tech/vet 223 - 54.390244%

Caretaker request 18 - 4.390244%

Not offered 63 - 15.365854%

Diaphragmatic hernia repairs

Routine 59 - 14.936709%

Discretion of tech/vet 205 - 51.898734%

Caretaker request 17 - 4.303797%

Not offered 114 - 28.860759%

For groups that test for FeLV, if a feral or community cat has a positive FeLV test, what is recommended? (Percentage: all survey respondents. Will not sum to 100% since some people used the "other" field to identify more than one common outcome, so a response was coded for two options.)

Retesting at a later date 91 - 16.04938%

Retesting on serum 72 - 12.69841%

Euthanasia if cat is symptomatic 48 - 8.465608%

Euthanasia regardless of symptoms 22 - 3.880071%

Transferred to a rescue/shelter 28 - 4.938272%

Return to site after sterilization 25 - 4.409171%

Other: Write-in answers grouped/summarized by authors. (Other percentages: all survey respondents)

Try to adopt/foster the cat 16 - 2.821869%

Transfer/relocate 16 - 2.821869%

Isolate the cat 5 - 0.8818342%

Monitor the cat 2 - 0.3527337%

For groups that test for FIV, if a feral or community cat has a positive FIV test, what is recommended? (Percentage: all survey respondents. Will not sum to 100% since some people used the "other" field to identify more than one common outcome, so a response was coded for two options.)

Retesting at a later date 31 - 5.467372%

Retesting on serum 7 - 1.234568%

Euthanasia if cat is symptomatic 68 - 11.99295%

Euthanasia regardless of symptoms 37 - 6.525573%

Transferred to a rescue/shelter 41 - 7.231041%

Return to site after sterilization 95 - 16.75485%

Other: Write-in answers grouped/summarized by authors. (Other percentages: all survey respondents)

Try to adopt/foster the cat 21 - 3.703704%

Transfer/relocate 5 - 0.8818342%

Isolate the cat 3 - 0.5291005%

Monitor the cat 6 - 1.058201%

Do you have a standardized location for vaccine injection sites? (Percentage: respondents to this question.)

No 130 - 33.078880%

Yes: rabies in the right rear leg 69 - 17.557252%

Yes: rabies in the right rear leg, FVRCP in the right front leg 165 - 41.984733%

Other 29 - 7.379135%

Does your organization re-trap cats in managed/cared-for colonies for rabies re-vaccination? (Percentage: respondents to this question.)

Always 7 - 1.635514%

Usually 21 - 4.906542%

Sometimes 76 - 17.757009%

Rarely 107 - 25.000000%

Never 217 - 50.700935%

How does your organization mark feral/community cats as sterilized? Rate each as performed always, on request, or never. (Percentage: respondents to question, percent is for each item.)

Ear tip (remove tip of ear)

Always 408 - 91.891892%

On request 27 - 6.081081%

Never 9 - 2.027027%

Ear notch (remove notch from ear)

Always 25 - 10.33058%

On request 26 - 10.74380%

Never 191 - 78.92562%

Tattoo in ventral midline abdominal region

Always 152 - 51.17845%

On request 44 - 14.81481%

Never 101 - 34.00673%

Ear tattoo

Always 15 - 6.172840%

On request 12 - 4.938272%

Never 216 - 88.888889%

Other: Write-in answers grouped/summarized by authors. (Other percentages: all survey respondents)

Microchip 2 - 0.3527337%

Tattoo females 3 - 0.5291005%

If you use ear tipping/notching of feral/community cats, on which side does it occur? (Percentage: respondents to this question.)

Left side 285 - 64.7727273%

Either ear, chosen based on convenience 72 - 16.3636364%

Right side 56 - 12.7272727%

Differs by sex: right female, left male 23 - 5.2272727%

Differs by sex: right male, left female 4 - 0.9090909%

For kitten spay/neuter, what type of lower limit do you use to determine if kittens can have surgery? (Percentage: respondents to this question.)

Minimum weight 264 - 58.149780%

Minimum age 16 - 3.524229%

Kitten must meet both a minimum age and minimum weight 174 - 38.325991%

If you use ONLY a minimum weight, what is that weight? (Percentage: respondents to this question.)

1.6 pounds 7 - 2.3890785%

1.7 pounds 2 - 0.6825939%

1.8 pounds 4 - 1.3651877%

1.9 pounds 1 - 0.3412969%

2.0 pounds 182 - 62.1160410%

2.1 pounds 5 - 1.7064846%

2.2 pounds 5 - 1.7064846%

2.3 pounds 5 - 1.7064846%

2.5 pounds 15 - 5.1194539%

2.7 pounds 1 - 0.3412969%

2.8 pounds 2 - 0.6825939%

3.0 pounds 45 - 15.3583618%

3.1 pounds 1 - 0.3412969%

3.5 pounds 3 - 1.0238908%

4.0 pounds 10 - 3.4129693%

4.5 pounds 1 - 0.3412969%

5.0 pounds 4 - 1.3651877%

If you use ONLY a minimum age, what is that age? (Percentage: respondents to this question.)

5 weeks 3 - 3.571429%

8 weeks / 2 months 35 - 41.666667%

9 weeks 2 - 2.380952%

10 weeks 5 - 5.952381%

11 weeks 2 - 2.380952%

12 weeks / 3 months 18 - 21.428571%

16 weeks / 4 months 12 - 14.285714%

20 weeks / 5 months 1 - 1.190476%

24 weeks / 6 months 2 - 2.380952%

Other 3 - 3.571429%

If a kitten must meet BOTH a minimum age and minimum weight, what are those two minimums? (Percentage: respondents to this question..)

2.2lb + 2 months 2 - 1.242236%

2.2lb + 3 months 1 - 0.621118%

2.5lb + 2 months 5 - 3.105590%

2.5lb + 3 months 1 - 0.621118%

2lb + 1.5 months 1 - 0.621118%

2lb + 2.5 months 3 - 1.863354%

2lb + 2 months 75 - 46.583851%

2lb + 3 months 11 - 6.832298%

2lb + 4 months 2 - 1.242236%

3.5lb + 3 months 1 - 0.621118%

3lb + 1.5 months 1 - 0.621118%

3lb + 2 months 3 - 1.863354%

3lb + 3.5 months 1 - 0.621118%

3lb + 3 months 32 - 19.875776%

3lb + 4 months 1 - 0.621118%

3lb + 5 months 1 - 0.621118%

4-5lb + 4-5 months 3 - 1.863354%

4lb + 1.5 months 1 - 0.621118%

4lb + 2.5 months 1 - 0.621118%

4lb + 3 months 2 - 1.242236%

4lb + 4 months 9 - 5.590062%

5lb + 3 months 1 - 0.621118%

Any weight + 6 months 2 - 1.242236%

After a routine surgery, what is your standard recovery process for all adult feline patients? Check all that apply. (Percentage: all survey respondents.  Will not sum to 100% since not everyone answered and some chose multiple answers.)

Administration of SQ fluids 46 - 8.112875%

Administration of SQ fluids - only in females 25 - 4.409171%

Corn syrup or dextrose applied along gumline/mouth 50 - 8.818342%

Checking patient’s temperature 95 - 16.75485%

Checking patient’s mucous membranes/capillary refill 107 - 18.87125%

Checking patient’s heart rate 119 - 20.98765%

Checking patient’s respiratory rate 130 - 22.92769%

Heat support (rice socks, heat discs, electric heating pads) 147 - 25.92593%

Two-stage recovery: first outside of a carrier/trap, then placed into a carrier/trap as cat regains consciousness 131- 23.10406%

Single-stage recovery: cat immediately placed in its carrier/trap after surgery 145 - 25.57319%

Small amount of food provided after patient is sternal and alert 209 - 36.86067%

Other: Write-in answers grouped/summarized by authors. (Other percentages: all survey respondents)

Sugar if needed 3 - 0.5291005%

Feed kittens 3 - 0.5291005%

Feed as needed 1 - 0.1763668%

Ingrown nail check 1 - 0.1763668%

Are perioperative antibiotics part of your routine spay/neuter procedure? (Percentage: respondents to this question.)

Yes 108 - 28.34646%

No 273 - 71.65354%

If you use antibiotics for any condition, what type of antibiotic do you use? Check all that apply. (Percentage: all survey respondents. Will not sum to 100% since not everyone answered and some chose multiple answers.)

Veterinary-formulated/marketed 379 - 66.84303%

Fish/aquarium-formulated/marketed 51 - 8.994709%

Human-formulated/marketed 46 - 8.112875%

Antibiotics available in feed stores for farm animals 33 - 5.820106%

If you are a private nonprofit or group, do you currently receive assistance (financial or supplies) from government public health or animal control programs? (Percentage: respondents to this question.)

Yes 43 - 9.907834%

No 382 - 88.018433%

Unsure 9 - 2.073733%

If you answered “yes” to the above question, what has been the form of that government assistance? Check all that apply. (Percentage: all survey respondents.)

General financial assistance (answer option) 18 - 3.174603%

Grants (combined from "Other") and General financial assistance 27 - 4.761905%

Humane traps and animal capture supplies 3 - 0.5291005%

Rabies vaccines for cats 7 - 1.234568%

FVRCP vaccines 5 - 0.8818342%

Drugs or surgical supplies/equipment 3 - 0.5291005%

Other: Write-in answers grouped/summarized by authors. (Other percentages: all survey respondents)

Grants 9 - 1.587302%

Animal control contracts 9 - 1.587302%

Spay/Neuter 7 - 1.234568%

Other 4 - 0.7054674%

Vouchers/reimbursements 3 - 0.5291005%

For what conditions are humane euthanasia recommended in feral/community cats? Check all that apply. (Percentage: all survey respondents. Will not sum to 100% since not everyone answered and some chose multiple answers.)

Single FeLV positive test if cat is symptomatic 135 - 23.80952%

Single FeLV positive test regardless of symptoms 80 - 14.10935%

Single FIV positive test if cat is symptomatic 85 - 14.99118%

Single FIV positive test regardless of symptoms 32 - 5.643739%

Single FeLV and FIV positive test if cat is symptomatic 103 - 18.16578%

Single FeLV and FIV positive test regardless of symptoms 45 - 7.936508%

Multiple FeLV positive tests if cat is symptomatic 99 - 17.46032%

Multiple FeLV positive tests regardless of symptoms 39 - 6.878307%

Multiple FIV positive tests if cat is symptomatic 53 - 9.347443%

Multiple FIV positive tests regardless of symptoms 15 - 2.645503%

Feline plasma cell pododermatitis 37 - 6.525573%

Feline stomatitis or severe dental disease 93 - 16.40212%

Masses suspected of being neoplastic 172 - 30.3351%

Severe respiratory disease 106 - 18.69489%

Loss of vision 81 - 14.28571%

Loss of limb 44 - 7.760141%

Signs of chronic illness (icteric, cachexia) 177 - 31.21693%

Heart murmur or arrhythmia 16 - 2.821869%

Cannot return to previous location 26 - 4.585538%

Other: Write-in answers grouped/summarized by authors. (Other percentages: all survey respondents)

Quality of life/suffering 99 - 17.46032%

Vet recommend 21 - 3.703704%

Trauma, pain, injury 12 - 2.116402%

Other 2 - 0.3527337%

**Data and Research**

If you collect data about feral/community cats, what do you use it for? Check all that apply. (Percentage: all survey respondents. Will not sum to 100% since not everyone answered and some chose multiple answers. One "Other" fill-in answer was tallied as the first answer choice.)

We don’t use it, we just collect it 37 - 6.525573%

Reports to our current funders 150 - 26.45503%

Applying for new grants and funding 248 - 43.73898%

Current/future campaigns aimed at changing laws 92 - 16.22575%

Challenging claims made by those who oppose TNR 117 - 20.63492%

Internal activity reporting 190 - 33.5097%

To modify or expand future trapping efforts 144 - 25.39683%

To periodically analyze progress and impact 171 - 30.15873%

Creating maps, graphs, and diagrams 107 - 18.87125%

Public presentations and documents 118 - 20.81129%

Other: Write-in answers. (Grouped/summarized by authors. Other percentages: all survey respondents)

Category: Admin/fundraising 23 - 4.056437%

Category: Medical 6 - 1.058201%

Category: Population impact 5 - 0.8818342%

Category: Activism/education/outreach 2 - 0.3527337%

Grouped by authors into categories. (Number of respondents in each group, some chose multiple answers.)

Administrative/fundraising 611

(Reports to our current funders, Applying for new grants and funding, Internal activity reporting, Other category: Admin/fundraising)

Activism/education/outreach 329

(Current/future campaigns, Challenging claims, Public presentations and documents, Other category: Activism/education/outreach)

Population impact 320

(To modify or expand future trapping efforts, To periodically analyze progress and impact, Other category: Population impact)

Creating maps, graphs, and diagrams 107

(Creating maps, graphs, and diagrams)

Just collect it 37

(We don’t use it, we just collect it)

Medical 6

(Other category: Medical)

What methods do you currently use to determine whether your program is effective at saving the lives of cats and/or reducing outdoor cat populations? Check all that apply. (Percentage: all survey respondents. Will not sum to 100% since not everyone answered and some chose multiple answers.)

Tracking shelter cat intake 177 - 31.21693%

Tracking shelter kitten intake 155 - 27.33686%

Tracking shelter cat euthanasia 126 - 22.22222%

Tracking cat nuisance calls made to animal control 72 - 12.69841%

Feedback from trappers/colony caretakers based on their judgement of cat numbers 268 - 47.26631%

Monitoring target cat populations at regular intervals to obtain a count or estimate of abundance or density 96 - 16.93122%

Monitoring target cat populations at regular intervals to obtain an estimate of sterilization rate 69 - 12.16931%

Monitoring target cat populations at regular intervals to obtain an estimate of proportion of kittens 79 - 13.93298%

Other: Write-in answers grouped/summarized by authors. (Other percentages: all survey respondents)

Category: Anecdotally 21 - 3.703704%

Category: Indirectly 11 - 1.940035%

Category: Ambiguous answer 4 - 0.7054674%

Category: Analytically 1 - 0.1763668%

Grouped by authors into categories. (Number of respondents in each group, some chose multiple answers.)

Indirectly 541

(Tracking shelter cat intake, Tracking shelter kitten intake, Tracking shelter cat euthanasia, Tracking cat nuisance calls)

Anecdotally 289

(Feedback from trappers/colony caretakers based on their judgement of cat numbers, Other category: Anecdotally)

Analytically 245

(Estimate of abundance or density, Estimate of sterilization rate, Estimate of proportion of kittens, Other category: Analytically)

Ambiguous 4

(Other category: Ambiguous answer)

Has your organization ever attempted to estimate the number of outdoor cats in a given area, and if so, using which method? Check all that apply. (Percentage: all survey respondents. Will not sum to 100% since not everyone answered and some chose multiple answers.)

No 240 - 53.45212%

Yes - Asking colony caretakers to count or estimate their cat numbers 174 - 30.68783%

Yes - Estimation using transect counts 17 - 2.998236%

Yes - Estimation using mark-recapture/mark-resight 19 - 3.35097%

Other: Write-in answers grouped/summarized by authors. (Other percentages: all survey respondents)

Category: Anecdotally 20 - 3.527337%

Category: Indirectly 5 - 0.8818342%

Category: Analytically 2 - 0.3527337%

Category: Ambiguous answer 3 - 0.5291005%

Category: Human population ratio 15 - 2.645503%

Grouped by authors into categories. (Number of respondents in each group.)

We don't 240

(No)

Anecdotally 194

(Asking colony caretakers to count or estimate their cat numbers)

Analytically 38

(Estimation using transect counts, Estimation using mark-recapture/mark-resight, Other category: Analytically)

Ratio 15

(Other category: Human population ratio)

Indirectly 5

(Other category: Indirectly)

Ambiguous 3

(Other category: Ambiguous answer)

Has your organization ever reached out to an academic or researcher for assistance with collecting data, analyzing data, or planning any aspect of your program? If so, please explain briefly, including the researcher's field/discipline. (Percentage: respondents to this question.)

No 405 - 90.200445%

Unsure 25 - 5.567929%

Yes 19 - 4.231626%

Grouped by authors, "yes" write-in explanations that provided a who and/or a why. (Percentage: all survey respondents.)

Who was contacted? Other 7 - 1.234568%

Who was contacted? Vet or academic 6 - 1.058201%

Who was contacted? Cat welfare organization 5 - 0.8818342%

Why? Planning S/N program 2 - 0.3527337%

Why? Quantifying cats 2 - 0.3527337%

Why? Data analysis 1 - 0.1763668%

Why? Making medical decisions 1 - 0.1763668%

Why? Changing laws 1 - 0.1763668%

Apart from this survey, has your group ever been contacted by an academic or researcher who wanted to work with you or collect data about your organization? If so, please explain briefly, including the researcher's field/discipline. (Percentage: respondents to this question.)

No 383 - 82.188841%

Unsure 39 - 8.369099%

Yes 44 - 9.442060%

Grouped by authors, "yes" write-in explanations that provided a who and/or a why. (Percentage: all survey respondents.)

Who contacted you? Cat welfare organization 13 - 2.292769%

Who contacted you? Students 9 - 1.587302%

Who contacted you? Academic 7 - 1.234568%

Who contacted you? Bird/wildlife group 1 - 0.1763668%

Why? Stats and data 7 - 1.234568%

Why? Animal welfare/behavior 7 - 1.234568%

Why? Bird/wildlife issues 5 - 0.8818342%

Why? Disease/medical 5 - 0.8818342%

Why? Biological samples 4 - 0.7054674%

Why? Quantifying cats 2 - 0.3527337%

Why? Business/marketing 2 - 0.3527337%

Why? Low-income outreach 2 - 0.3527337%

Why? Surveys 2 - 0.3527337%

Why? Human-animal bond 1 - 0.1763668%

Would you utilize expert assistance in designing and interpreting your data collection if such assistance were available? (Percentage: respondents to this question.)

Yes, and we would be willing to pay a reasonable fee for such assistance 21 - 4.545455%

Yes, but only if such assistance is provided without cost 235 - 50.865801%

Unsure 138 - 29.870130%

No 68 - 14.718615%

Would you be interested in collaborating with researchers if the aim was to improve the welfare of feral/community cats? (Percentage: respondents to this question.)

Definitely 241 - 51.605996%

Possibly 159 - 34.047109%

Unsure 40 - 8.565310%

Unlikely 17 - 3.640257%

No 10 - 2.141328%

Would you be interested in collaborating with researchers if the aim was to improve the health/welfare of owned cats? (Percentage: respondents to this question.)

Definitely 217 - 46.466809%

Possibly 162 - 34.689507%

Unsure 42 - 8.993576%

Unlikely 31 - 6.638116%

No 15 - 3.211991%

Would you be interested in collaborating with researchers if the aim is to study public health issues? (Percentage: respondents to this question.)

Definitely 115 - 24.67811%

Possibly 173 - 37.12446%

Unsure 75 - 16.09442%

Unlikely 51 - 10.94421%

No 52 - 11.15880%

Would you be interested in collaborating with researchers if the aim was to study cat impacts on birds and wildlife? (Percentage: respondents to this question.)

Definitely 100 - 21.45923%

Possibly 156 - 33.47639%

Unsure 75 - 16.09442%

Unlikely 78 - 16.73820%

No 57 - 12.23176%

**Bird and Wildlife Issues**

Does your organization have an official position (such as a statement on your website) about the impact of outdoor cats on birds and wildlife, and if so, which of the given options is closest to that organization-level position? (Percentage: respondents to this question.)

We have no official position/statement about this topic 375 - 83.518931%

Cats often have a serious impact on birds and / or other wildlife 11 - 2.449889%

Cats may have a serious impact on birds or other wildlife in some places, but little or no serious impact in other places 25 - 5.567929%

Cats rarely or never have a serious impact on birds or other wildlife 38 - 8.463252%

Does your organization have an official position (such as a statement on your website) about how TNR programs change the impact of outdoor cats on birds and wildlife, and if so, which of the given options is closest to that organization-level position? (Percentage: respondents to this question.)

We have no official position/statement about this topic 343 - 75.7174393%

TNR programs generally reduce these impacts 90 - 19.8675497%

TNR programs generally do not change these impacts 5 - 1.1037528%

TNR programs generally increase these impacts 1 - 0.2207506%

TNR programs have impacts that vary from place to place 14 - 3.0905077%

How would the current relationship between “feral/community cat people” and “wildlife/bird people” in your area best be described? (Percentage: respondents to this question.)

Public/overt conflict occurring 33 - 7.221007%

Some tension between groups 161 - 35.229759%

Neutral or no interactions 196 - 42.888403%

Some efforts being made towards bridge-building 22 - 4.814004%

Active collaboration and working towards shared goals 5 - 1.094092%

We serve many locations, each is different 40 - 8.752735%

Have positive collaborations occurred between “feral/community cat people” and “wildlife/bird people,” and if so, how did that process initially start? (Percentage: respondents to this question.)

No collaborations 328 - 79.6116505%

Feral/community cat organizations formally reached out to wildlife/bird organizations 15 - 3.6407767%

Wildlife/bird organizations formally reached out to feral/community cat organizations 1 - 0.2427184%

Individuals involved in feral cat issues reached out to individuals they know who are involved in wildlife/bird issues 33 - 8.0097087%

Individuals involved in wildlife/bird issues reached out to individuals they know who are involved in feral cat issues 5 - 1.2135922%

Working together grew out of tension or public conflict 15 - 3.6407767%

Other: Write-in answers grouped/summarized by authors. 15 - 3.6407767%

Other 9

Attempts failed 4

Government 1

We do cats and birds/wildlife 1
