# Supplementary material for: “State of the Mewnion”: Practices of Feral Cat Care and Advocacy Organizations in the United States
Source: Front Vet Sci. 2021 Dec 14;8:791134. doi: 10.3389/fvets.2021.791134 (PMC8712445; doi:10.3389/fvets.2021.791134)
Supplement: Supplementary file 2 [file Table_2.DOCX]

**Instructions Summary On the Top of Each Page**

Because this survey covers many areas, the best person to complete the survey on behalf of their organization is someone familiar with the workings of the group on every level, such as a manager or Executive Director, with input from a veterinarian or Medical Director if the group has one. From January 15 to March 15 while the survey is open, you can go back and edit your answers so long as the survey is attempted again from the same computer and same internet browser. This will help if you need two people complete the survey, such as if you want one person answering general questions and a vet or vet tech answering the medical questions. Please make sure that this survey is submitted only once on behalf of each organization. If you are having any technical problems filling out this survey, contact Sabrina Aeluro at aeluro@uw.edu.

This survey is lengthy so we can ask questions about a wide range of possible organization functions. You may find that only a small number of questions apply to your group. Your responses are very much appreciated, whatever the size and scope of your organization.

**Organization Basics - These Questions Are Required**

Where is your organization located/based? Remember, this survey is for United States-based organizations only.)

[Dropdown of states/US territories]

What is the geographical area that your organization serves most of the time?

Neighborhood/development

City/town

Statewide

Multi-state regional

National

International, based in the US

Which areas does your organization serve? Check all that apply.

Urban

Suburban

Rural

What is the approximate proportion of animals that your organization serves/advocates for which are feral/community cats? (Including kittens that are the offspring of such cats.)

¼ or less

¼ to ½

½ to ¾

¾ or more

Does your organization have its own 501(c)3 status?

Yes

No

No, but we can receive tax deductible donations through another organization

Does your group have a brick and mortar facility? (Apart from operating out of a person’s home.)

Yes

Yes, as a part of a shared space with another project or within a larger organization

No

If you provide services or caretaking, what is the approximate number of feral/community cats that you serve per year?

Fewer than 99

100-499

500-999

1000-2499

2499-4999

5000-9999

10000-14999

15000-19999

20000 or more

Is your group a project/club run by veterinary students?

Yes

No

What is the number of paid employees in your group? (Or, if your organization does some feral/community work alongside other functions, what is the number of paid employees just within your feral/community cat section?)

0

1-4

5-9

10-19

20 or more

What is the number of active volunteers in your group? (Or, if your organization does some feral/community work alongside other functions, what is the number of volunteers just within your feral/community cat section?)

0

1-9

10-19

20-49

50-99

100 or more

What does your organization do? Categorize each as a primary, secondary, or rare function, or not offered.

Direct feeding and colony care for feral/community cats

Direct trapping of feral/community cats for TNR

Coordinating volunteers who are trapping feral/community cats for TNR

Training or holding workshops for caretakers and trappers

We operate our own clinic focused on feral/community cat care

FREE sterilization/vaccination/basic medical care for feral/community cats

LOW-COST sterilization/vaccination/basic medical care for feral/community cats

FREE sterilization/vaccination/basic medical care for owned cats from low-income households

LOW-COST sterilization/vaccination/basic medical care for owned cats from low-income households

FREE sterilization/vaccination/basic medical care for cats from animal shelters/rescues

LOW-COST sterilization/vaccination/basic medical care for cats from animal shelters/rescues

Socializing/fostering adult feral/community cats for adoption

Socializing/fostering kittens from feral/community cats for adoption

Running a sanctuary for feral/community cats

We are an animal shelter with a return-to-field program for feral/community cats

Operating a “working cats” program for urban/suburban mousers

Operating a “barn cats” program for rural mousers

Providing disaster relief for feral/community cats as needed

Engaging in organization-level training and mentorship to other feral/community cat groups

Public education and awareness raising around feral/community cat welfare issues

Resource sharing/community building for those already working to help feral/community cats

Campaigning for law and policy changes around feral/community cats

Coalition-building between feral/community cat advocates and bird/conservation advocates

Providing grants and funding organizations doing feral/community cat work

Training of volunteers and caregivers regarding trapping, colony care, and best practices

Participating in research projects

**Administration and Policy**

Is TNR explicitly allowed or endorsed by local laws and animal control ordinances where you operate?

Yes

No

Unsure

It varies, we operate across many jurisdictions

If TNR is not explicitly legal in your area, are there local laws that could be used, or are actively being enforced, to prohibit or limit feral/community cat care, feeding, or TNR? Rate each item.

Could Be Used Actively Enforced Unknown

Laws against feeding

Laws defining outdoor cat feeders as the cat’s owner

Pet licensing laws

Mandatory “stray” holding periods

Pet limits

Animal control of “nuisance” animals

Required colony registration

Mandatory spay/neuter requirements

Microchipping requirements

Leash laws which include cats

Abandonment laws

Laws restricting veterinarians’ abilities to provide free/low-cost services

Other: please describe briefly [____________]

Has your organization consulted with an attorney regarding legal problems that could arise from your work?

Yes, at some point in the past

Yes, on an ongoing basis

No

Unsure

How would the current relationship between feral/community cat advocates and animal control authorities in your area best be described?

Public/overt conflict occurring

Some tension between groups

Neutral or no interactions

Some efforts being made towards bridge-building

Active collaboration and working towards shared goals

We serve many locations, each is different

We are the animal control authorities in our area

Does your organization have a locally-focused online discussion group or email list where feral/community cat advocates can ask questions, share resources, seek assistance, and support one another?

Yes

No

Does your organization currently have at least one declared goal that includes both a measurable value and a timeframe? Such as, “reduce the outdoor cat population of our town 25% by 2025” or “provide 1000 free spay/neuter surgeries every year.”

Yes

No

If feral/community cats is one part of what your organization does, are there reasons why you don't serve more feral/community cats? Check all that apply.

We are a comprehensive animal program that fulfills many roles

We would do more with feral/community cats if we had specific grants/funding

We don’t have the proper facilities or equipment

Our staff doesn’t have the proper training

Concern about possible injuries to staff and volunteers

There is an alternative for feral/community cat care in our area

Our organization has a policy that prevents (more) care of feral/community cats

Unsure

Other: please explain briefly [___________]

People sometimes discuss “feral cats,” “stray cats,” “barn cats,” or “community cats” as separate categories, using their own definitions of these groups. Does your organization have policies or priorities that differentiate between classifications of outdoor, free-roaming cats?

Yes

No

Unsure

What resources (books, websites, blogs, Facebook groups, etc.) does your organization regularly use and trust for updates, information, and news about feral/community cat issues?

[_____________]

**Environmental, Human, and Public Health**

Do you have a formal process for staff or volunteers who receive bites or other injuries from feral/community cats?

Yes

No

Does your organization maintain insurance for staff and volunteers to cover medical care for injuries sustained during work with feral/community cats?

Yes

No

Does your organization provide staff and/or volunteers with mental health care resources, such as information on compassion fatigue, support groups for animal welfare workers, suicide and crisis hotlines, or referrals to mental health providers?

Yes

No

Are you seeing health issues in feral/community cats that are suspected as being caused by exposures to toxins or environmental contaminants?

No

Unknown

Yes: please explain briefly [___________]

**Caretaking, Trapping, and Release**

Does your group provide feeders/caretakers with any official recommendations or training for best practices, such as the amount of food to provide, information on making outdoor cat shelters, monitoring cat health, dealing with conflicts with neighbors, or how to trap cats?

Yes

No

Are colonies or colony caretakers in your service area required by law to be registered in some way?

Yes

No

Unsure

Regardless of whether registration is required by law, approximately what proportion of colonies or colony caretakers in your service area do you estimate are actually registered?

¼ or less

¼ to ½

½ to ¾

¾ or more

If you do register colonies or caretakers, where is this information stored?

With a government office

With a private nonprofit/individual

Both government and private

Unsure

If you do not always register colonies or caretakers, why not? Check all that apply.

Some caretakers have refused

We believe caretakers might be resistant

Feeding/TNR is illegal in our area

We were advised by an attorney to not document colonies/caretakers

We lack the tools or technical resources to maintain a registry

We lack the time or personnel to maintain a registry

We don’t see a reason to register colonies/caretakers

We are intending on implementing a registry (or better registry) soon

Other: please describe briefly [____________]

For groups trapping feral/community cats for sterilization, how does your organization decide where to trap? Rate each reason as higher priority, lower priority, or not a factor used when trapping.

Complaints from the public about number of cats in a location

Requests from colony caretakers

Places located conveniently for trappers (such as near their homes)

Locations that are safe for trappers to work

Evenly distributing efforts to provide some sterilizations to as many caretakers as possible

Concentrating efforts in smaller areas to get high sterilization coverage of some areas

Trapping in one area or colony until all cats are caught and sterilized

Providing TNR services to low-income neighborhoods

Locations from which many cats are entering the shelter/animal control system

Areas where cats are suspected to pose a risk to birds and wildlife

Based on funding/grants that specify where we will provide services

Trapping for TNR and relocation to protect cats at risk of harm

Other: please describe briefly [____________]

For kittens (born to feral/community cats) under 2 months of age, do you remove them from the outdoors for fostering, socialization, and adoption?

Always

Usually

Sometimes

Rarely

Never

For kittens (born to feral/community cats) between 2 and 3 months of age, do you remove them from the outdoors for fostering, socialization, and adoption?

Always

Usually

Sometimes

Rarely

Never

If you do adoptions of socialized feral/community cats or kittens born to feral/community cats, does your group’s adoption information have a position on whether they should be kept as indoor-only animals?  (This is regarding adoptions of socialized cats/kittens as traditional pets, not barn cat/working cat programs.)

We require that adopted cats/kittens be indoor-only

We recommend that adopted cats/kittens be indoor-only

We require that adopted cats/kittens be allowed both indoors and outdoors

We recommend that adopted cats/kittens be allowed both indoors and outdoors

We have no position on where adopters keep their cats/kittens

Other

After a routine neuter with no complications, what is your policy on what happens to male cats?

Always released on the same day as surgery

Held overnight and then released

Held for 2 nights and then released

Held for 3 nights and then released

Other

After a routine spay with no complications, what is your policy on what happens to female cats?

Always released on the same day as surgery

Held overnight and then released

Held for 2 nights and then released

Held for 3 nights and then released

Other

Does your organization routinely recommend or use any supplements or alternative medicine products with feral/community cats, and if so, which type? Check all that apply.

No supplements/alternative medicine products

Probiotics (such as FortiFlora™)

Rescue Remedy®

Homeopathic products

Herbal products

Glucosamine

Feliway®

**Clinical and Medical Issues - Best Completed by a Vet/Vet Tech**

What, if any, published guidelines do you use in shaping your own TNR and medical practices? Check all that apply.

Association of Shelter Veterinarians Guidelines for Spay-Neuter Programs

American Association of Feline Practitioners Practice Guidelines

Neighborhood Cats TNR Handbook

Community TNR: Tactics and Tools by Bryan Kortis

Best Friends Community Cat Programs Handbook

Alley Cat Allies/ASPCA/Mayor’s Alliance for NYC’s Animals Guide to Trap-Neuter-Return and Colony Care

ASPCA’s Special Considerations for Community Cats at Spay/Neuter Clinics

Alley Cat Allies Veterinary Resource Center

ISFM Guidelines on Population Management and Welfare of Unowned Domestic Cats

ICAM Coalition Humane Cat Population Management Guidance

Other: please list [____________]

If your group traps cats but does not operate a clinic, approximately how far must animals be transported to reach your nearest provider of sterilization services for feral/community cats?

Under 30 minutes by car

30-60 minutes by car

60-90 minutes by car

90-120 minutes by car

2-4 hours by car

4-6 hours by car

6 or more hours by car

Whether or not you operate your own clinic, approximately how far away is the next-nearest provider of sterilization services for feral/community cats?

Under 30 minutes by car

30-60 minutes by car

60-90 minutes by car

90-120 minutes by car

2-4 hours by car

4-6 hours by car

6 or more hours by car

Unknown

If you provide or facilitate sterilization and veterinary services, are your fees different for cats reported as owned versus cats reported as being feral/community cats?

Yes

No

If you provide free or discounted services to low-income caretakers and trappers of feral/community cats, do you have a stated cut-off for what qualifies as “low-income”?

Yes

No

If you answered “yes” above, do you require documentation of low-income status, such as with a pay stub or tax return, or a person’s qualification for federal assistance programs like Medicare?

Yes

Decided on a case-by-case basis

No

If your organization has a standard required fee or suggested donation to pay for veterinary services for feral/community cats, what is that amount in US dollars and what does it include? (While you may offer additional services, please just consider these six scenarios.)

For routine female spay $[___]

For routine male neuter $[___]

For routine female spay, plus rabies vaccine $[___]

For routine male neuter, plus rabies vaccine $[___]

For routine female spay, plus rabies and FVRCP vaccines $[___]

For routine male neuter, plus rabies and FVRCP vaccines $[___]

Are feral/community cats scanned for microchips during your TNR process?

Always

Sometimes

Never

If you microchip feral/community cats, are the microchips registered somewhere? Check all that apply.

Yes, registered with a standard pet microchip company’s database

Yes, registered with a rescue group

Yes, registered with local animal control

No, the chip numbers are just for the caretaker’s records

Other: please describe briefly [____________]

What clinical services do you provide to feral/community cats? Categorize each as routine (done to every animal), done at the discretion of a veterinarian or vet tech, provided if requested by a caretaker, or not offered.

Routine Discretion of tech/vet Caretaker request Not offered

Flea/ectoparasite treatment

De-worming/endoparsite treatment

Rabies vaccination

FVRCP vaccination

FeLV vaccination

Microchipping

Meloxicam or other injectable pain relief

Fluids

FIV testing

FeLV testing

Cleaning of wounds/abscesses

Extraction of diseased teeth

Dental cleanings

Enucleation

Entropian repair

Tail amputation

Limb amputation

Declaw of embedded claw

Umbilical hernia repairs

Diaphragmatic hernia repairs

Other: please list briefly [_______]

Do you have a standardized location for vaccine injection sites?

No

Yes: rabies in the right rear leg

Yes: rabies in the right rear leg, FVRCP in the right front leg

Other: please describe briefly [____________]

For groups that test for FeLV, if a feral or community cat has a positive FeLV test, what is recommended?

Retesting at a later date

Retesting on serum

Euthanasia if cat is symptomatic

Euthanasia regardless of symptoms

Transferred to a rescue/shelter

Return to site after sterilization

Other: please explain briefly [___________]

For groups that test for FIV, if a feral or community cat has a positive FIV test, what is recommended?

Retesting at a later date

Retesting on serum

Euthanasia if cat is symptomatic

Euthanasia regardless of symptoms

Transferred to a rescue/shelter

Return to site after sterilization

Other: please explain briefly [___________]

How does your organization mark feral/community cats as sterilized? Rate each as performed always, on request, or never.

Always On Request Never

Ear tip (remove tip of ear)

Ear notch (remove notch from ear)

Tattoo in ventral midline abdominal region

Ear tattoo

Other, please specify [__________]

If you use ear tipping/notching of feral/community cats, on which side does it occur?

Either ear, chosen based on convenience

Right side

Left side

Differs by sex: right female, left male

Differs by sex: right male, left female

For kitten spay/neuter, what type of lower limit do you use to determine if kittens can have surgery?

Minimum weight

Minimum age

Kitten must meet both a minimum age and minimum weight

If you use ONLY a minimum weight, what is that weight?

1.6 pounds

…

4.9 pounds

5.0 pounds

Other, please specify weight in pounds

If you use ONLY a minimum age, what is that age?

5 weeks

6 weeks

7 weeks

8 weeks / 2 months

9 weeks

10 weeks

11 weeks

12 weeks / 3 months

13 weeks

14 weeks

15 weeks

16 weeks / 4 months

Other, please specify [__________]

If a kitten must meet BOTH a minimum age and minimum weight, what are those two minimums?

[__________]

For what conditions are humane euthanasia recommended in feral/community cats? Check all that apply.

Single FeLV positive test if cat is symptomatic

Single FeLV positive test regardless of symptoms

Single FIV positive test if cat is symptomatic

Single FIV positive test regardless of symptoms

Single FeLV and FIV positive test if cat is symptomatic

Single FeLV and FIV positive test regardless of symptoms

Multiple FeLV positive tests if cat is symptomatic

Multiple FeLV positive tests regardless of symptoms

Multiple FIV positive tests if cat is symptomatic

Multiple FIV positive tests regardless of symptoms

Feline plasma cell pododermatitis

Feline stomatitis or severe dental disease

Masses suspected of being neoplastic

Severe respiratory disease

Loss of vision

Loss of limb

Signs of chronic illness (icteric, cachexia)

Heart murmur or arrhythmia

Cannot return to previous location

Other: please explain briefly [___________]

After a routine surgery, what is your standard recovery process for all adult feline patients? Check all that apply.

Administration of SQ fluids

Administration of SQ fluids - only in females

Corn syrup or dextrose applied along gumline/mouth

Checking patient’s temperature

Checking patient’s mucous membranes/capillary refill

Checking patient’s heart rate

Checking patient’s respiratory rate

Heat support (rice socks, heat discs, electric heating pads)

Two-stage recovery: first outside of a carrier/trap, then placed into a carrier/trap as cat regains consciousness

Single-stage recovery: cat immediately placed in its carrier/trap after surgery

Small amount of food provided after patient is sternal and alert

Other: please explain briefly [___________]

Are perioperative antibiotics part of your routine spay/neuter procedure?

Yes

No

Does your organization re-trap cats in managed/cared-for colonies for rabies re-vaccination?

Always

Usually

Sometimes

Rarely

Never

If you use antibiotics for any condition, what type of antibiotic do you use? Check all that apply.

Veterinary-formulated/marketed

Human-formulated/marketed

Fish/aquarium-formulated/marketed

Antibiotics available in feed stores for farm animals

If you are a private nonprofit or group, do you currently receive assistance (financial or supplies) from government public health or animal control programs?

Yes

No

Unsure

If you answered “yes” to the above question, what has been the form of that government assistance? Check all that apply.

General financial assistance

Humane traps and animal capture supplies

Rabies vaccines for cats

FVRCP vaccines

Drugs or surgical supplies/equipment

Other: please describe briefly [____________]

**Statistics and Research**

If you collect data about feral/community cats, what do you use it for? Check all that apply.

We don’t use it, we just collect it

Reports to our current funders

Applying for new grants and funding

Current/future campaigns aimed at changing laws

Challenging claims made by those who oppose TNR

Internal activity reporting

To modify or expand future trapping efforts

To periodically analyze progress and impact

Creating maps, graphs, and diagrams

Public presentations and documents

Other: please explain briefly [___________]

What methods do you currently use to determine whether your program is effective at saving the lives of cats and/or reducing outdoor cat populations? Check all that apply.

Tracking shelter cat intake

Tracking shelter kitten intake

Tracking shelter cat euthanasia

Tracking cat nuisance calls made to animal control

Feedback from trappers/colony caretakers based on their judgement of cat numbers

Monitoring target cat populations at regular intervals to obtain a count or estimate of abundance or density

Monitoring target cat populations at regular intervals to obtain an estimate of sterilization rate

Monitoring target cat populations at regular intervals to obtain an estimate of proportion of kittens

Other: please explain briefly [___________]

Has your organization ever attempted to estimate the number of outdoor cats in a given area, and if so, using which method? Check all that apply.

No

Yes - Asking colony caretakers to count or estimate their cat numbers

Yes - Estimation using transect counts

Yes - Estimation using mark-recapture/mark-resight

Yes - Other method, please explain briefly [___________]

Has your organization ever reached out to an academic or researcher for assistance with collecting data, analyzing data, or planning any aspect of your program? If so, please explain briefly, including the researcher's field/discipline.

No

Unsure

Yes: [____________]

Apart from this survey, has your group ever been contacted by an academic or researcher who wanted to work with you or collect data about your organization? If so, please explain briefly, including the researcher's field/discipline.

No

Unsure

Yes: [____________]

Would you utilize expert assistance in designing and interpreting your data collection if such assistance were available?

Yes, and we would be willing to pay a reasonable fee for such assistance

Yes, but only if such assistance is provided without cost

Unsure

No

Would you be interested in collaborating with researchers if the aim was to improve the welfare of feral/community cats?

Definitely

Possibly

Unsure

Unlikely

No

Would you be interested in collaborating with researchers if the aim was to improve the health/welfare of owned cats?

Definitely

Possibly

Unsure

Unlikely

No

Would you be interested in collaborating with researchers if the aim was to study cat impacts on birds and wildlife?

Definitely

Possibly

Unsure

Unlikely

No

Would you be interested in collaborating with researchers if the aim is to study public health issues?

Definitely

Possibly

Unsure

Unlikely

No

**Bird/Wildlife Conflict Topics**

How would the current relationship between “feral/community cat people” and “wildlife/bird people” in your area best be described?

Public/overt conflict occurring

Some tension between groups

Neutral or no interactions

Some efforts being made towards bridge-building

Active collaboration and working towards shared goals

We serve many locations, each is different

Have positive collaborations occurred between “feral/community cat people” and “wildlife/bird people,” and if so, how did that process initially start?

No collaborations

Feral/community cat organizations formally reached out to wildlife/bird organizations

Wildlife/bird organizations formally reached out to feral/community cat organizations

Individuals involved in feral cat issues reached out to individuals they know who are involved in wildlife/bird issues

Individuals involved in wildlife/bird issues reached out to individuals they know who are involved in feral cat issues

Working together grew out of tension or public conflict

Other: please explain briefly [___________]

Does your organization have an official position (such as a statement on your website) about the impact of outdoor cats on birds and wildlife, and if so, which of the given options is closest to that organization-level position?

We have no official position/statement about this topic

Cats often have a serious impact on birds and / or other wildlife

Cats may have a serious impact on birds or other wildlife in some places, but little or no serious impact in other places

Cats rarely or never have a serious impact on birds or other wildlife

Does your organization have an official position (such as a statement on your website) about how TNR programs change the impact of outdoor cats on birds and wildlife, and if so, which of the given options is closest to that organization-level position?

We have no official position/statement about this topic

TNR programs generally reduce these impacts

TNR programs generally do not change these impacts

TNR programs generally increase these impacts

TNR programs have impacts that vary from place to place

**Optional Email Collection**

If you would like to receive a copy of the results of this study later in 2018, please enter you email address below.

Box

If you would like to be contacted about future feral/community cat related surveys, please enter your email address below.

Box
